# Acute complications and mortality in hospitalized patients with coronavirus disease 2019: a systematic review and meta-analysis

**DOI:** 10.1186/s13054-020-03022-1

**Published:** 2020-07-02

**Authors:** Nicola Potere, Emanuele Valeriani, Matteo Candeloro, Marco Tana, Ettore Porreca, Antonio Abbate, Silvia Spoto, Anne W. S. Rutjes, Marcello Di Nisio

**Affiliations:** 1 Department of Medical, Oral and Biotechnological Sciences, “G. D’Annunzio” University, Via Dei Vestini 31, 66100 Chieti, Italy; 2 Department of Medicine and Ageing Sciences, “G. d’Annunzio” University, Chieti-Pescara, Italy; 3 VCU Heart Center, Virginia Commonwealth University, Richmond, VA USA; 4 Internal Medicine Department, University Hospital Campus Bio-Medico, Rome, Italy; 5 Institute of Social and Preventive Medicine (ISPM), University of Bern, Bern, Switzerland; 6 Department of Vascular Medicine, Academic Medical Center, Amsterdam, The Netherlands

**Keywords:** Adult respiratory distress syndrome, Coronavirus, COVID-19, Oxygen inhalation therapy, SARS virus

## Abstract

**Background:**

The incidence of acute complications and mortality associated with COVID-19 remains poorly characterized. The aims of this systematic review and meta-analysis were to summarize the evidence on clinically relevant outcomes in hospitalized patients with COVID-19.

**Methods:**

MEDLINE, EMBASE, PubMed, and medRxiv were searched up to April 20, 2020, for studies including hospitalized symptomatic adult patients with laboratory-confirmed COVID-19. The primary outcomes were all-cause mortality and acute respiratory distress syndrome (ARDS). The secondary outcomes included acute cardiac or kidney injury, shock, coagulopathy, and venous thromboembolism. The main analysis was based on data from peer-reviewed studies. Summary estimates and the corresponding 95% prediction intervals (PIs) were obtained through meta-analyses.

**Results:**

A total of 44 peer-reviewed studies with 14,866 COVID-19 patients were included. In general, risk of bias was high. All-cause mortality was 10% overall (95% PI, 2 to 39%; 1687/14203 patients; 43 studies), 34% in patients admitted to intensive care units (95% PI, 8 to 76%; 659/2368 patients; 10 studies), 83% in patients requiring invasive ventilation (95% PI, 1 to 100%; 180/220 patients; 6 studies), and 75% in patients who developed ARDS (95% PI, 35 to 94%; 339/455 patients; 11 studies). On average, ARDS occurred in 14% of patients (95% PI, 2 to 59%; 999/6322 patients; 23 studies), acute cardiac injury in 15% (95% PI, 5 to 38%; 452/2389 patients; 10 studies), venous thromboembolism in 15% (95% PI, 0 to 100%; patients; 3 studies), acute kidney injury in 6% (95% PI, 1 to 41%; 318/4682 patients; 15 studies), coagulopathy in 6% (95% PI, 1 to 39%; 223/3370 patients; 9 studies), and shock in 3% (95% PI, 0 to 61%; 203/4309 patients; 13 studies).

**Conclusions:**

Mortality was very high in critically ill patients based on very low-quality evidence due to striking heterogeneity and risk of bias. The incidence of clinically relevant outcomes was substantial, although reported by only one third of the studies suggesting considerable underreporting.

**Trial registration:**

PROSPERO registration ID for this study is CRD42020177243 (https://www.crd.york.ac.uk/prospero/display_record.php?RecordID=177243).

## Background

Clinical manifestations of *Coronavirus* disease 2019 (COVID-19) range from mild cases to severe pneumonia, which may be complicated by an exaggerated systemic inflammatory response leading to acute respiratory distress syndrome (ARDS), multiple organ failure, and death [1–3]. The largest report on COVID-19 from the Chinese Center for Disease Control and Prevention showed that among 44,672 confirmed cases, 80.9% were mild, 13.9% severe, and 4.7% critical. The overall case-fatality rate was 2.3% with values as high as 49% in critical patients with respiratory failure, septic shock, or multiple organ failure [4].

Since the start of the outbreak, observational studies have extensively described the most prevalent clinical, laboratory, and radiological presentations of COVID-19. However, the clinical course of patients with COVID-19 remains poorly characterized, with heterogeneous and discordant incidence of in-hospital complications and mortality [5–7].

The aims of this systematic review and meta-analysis were to evaluate current evidence on clinically relevant outcomes in hospitalized symptomatic adult patients with COVID-19 and identify factors which may predict worse prognosis.

## Methods

This study-level systematic review and meta-analysis was performed following the Preferred Reporting Items for Systematic reviews and Meta-analysis (PRISMA) guidelines [8].

The PROSPERO registration ID is CRD42020177243.

### Databases search and study selection

MEDLINE, EMBASE (Ovid-SP), and PubMed were searched from inception up to April 20, 2020, for observational studies and randomized controlled trials (RCTs) in English language which included symptomatic adult patients with COVID-19. This search was complemented with the screening of medRxiv, a free online archive for complete but unpublished manuscripts (preprints) in the health sciences (https://www.medrxiv.org/content/about-medrxiv), until April 20, 2020. The preprints available in medRxiv represent preliminary reports of studies that have not been peer-reviewed yet; thus, they may help to provide a comprehensive picture of the evidence that may be published in future, but they should not be relied on to guide clinical practice. The complete search strategy is given in Additional file 1: Tables S1 and S2.

Two authors independently reviewed titles and abstracts identified from the search to select studies which met the following inclusion criteria: (i) observational study or RCT enrolling ≥ 50 symptomatic outpatients or hospitalized patients, (ii) laboratory-confirmed COVID-19 defined by a positive result on a reverse-transcriptase-polymerase-chain-reaction assay of a nasopharyngeal and oropharyngeal swabs or a sputum specimen, and (iii) at least one of the primary outcomes reported by the study. Case reports and case series with less than 50 patients were excluded as they may observe no events due to the small size. Full-text screening was done independently by the same authors, and any disagreement was resolved through discussion or involving a third review author.

### Data extraction and quality assessment

Two review authors independently extracted data from the included studies. Any disagreement was resolved by consensus between the two review authors or by involving a third review author. The following data were extracted: study characteristics (e.g., study design, health-care setting), patients’ characteristics (e.g., age, sex), presence of comorbidities (e.g., respiratory system disease, chronic kidney disease, cardiovascular disease, diabetes, hypertension, malignancy), severity of COVID-19 disease at study entry, treatment (e.g., antivirals, antibiotic therapy, invasive ventilation and non-invasive oxygen support), and clinical outcomes. We extracted the number of patients with severe COVID-19 at study entry and recorded for each study whether internationally accepted criteria or authors’ own definitions were used [9–11].

The methodological quality was evaluated using the methodological index for non-randomized studies (MINORS) tool for observational studies and the Cochrane tool for RCTs [12, 13]. The MINORS tool evaluates eight study quality items and classifies them as adequate, inadequate, or unclear [12]. The MINORS’ item on blinded evaluation was deemed adequate for ARDS if an external independent adjudication committee was involved. The item related to the endpoint definition was considered adequate if unambiguous criteria were used to define the review outcomes. Follow-up duration was considered adequate if all included patients were followed up until hospital discharge or death.

### Study outcomes

The primary outcomes were all-cause mortality and ARDS. The secondary outcomes included shock, acute kidney injury, acute cardiac injury, coagulopathy, venous thromboembolism, and major bleeding events.

It was anticipated that outcome definitions varied across included studies. This aspect was considered in the evaluation of the study quality item regarding the appropriateness of the criteria used to define the primary outcomes. Standard criteria to diagnose the outcomes of interest included the following: ARDS diagnosed according to the Berlin definition [14]; shock defined as persisting hypotension despite volume resuscitation, requiring vasopressors to maintain mean arterial pressure ≥ 65 mmHg and serum lactate level > 2 mmol/L [15]; acute kidney injury diagnosed as an increase in serum creatinine by ≥ 0.3 mg/dl within 48 h, ≥ 1.5 times from baseline within 7 days, or urine volume < 0.5 ml/kg/h for 6 h [16]; acute myocardial injury diagnosed when there was a rise and/or fall of cardiac troponin values with at least one value above the 99th percentile of the upper reference limit [17]; coagulopathy including the development of disseminated intravascular coagulopathy defined according to the International Society of Thrombosis and Hemostasis [18], or abnormal values of specific coagulation markers [19]; venous thromboembolism including fatal or non-fatal pulmonary embolism and deep vein thrombosis; and major bleeding defined according to the criteria of the International Society of Thrombosis and Hemostasis [20].

### Statistical analysis

The main analysis was based on data extracted from peer-reviewed studies that were retrieved from MEDLINE, EMBASE, and PubMed databases. The analyses performed on not peer-reviewed studies retrieved from medRxiv were considered exploratory.

For descriptive purposes, we used the mean and standard deviation for continuous variables or the median and range, where appropriate. Categorical variables were described as counts and percentages. Summary estimates and the corresponding 95% prediction intervals (PIs) and 95% confidence intervals (CIs) were obtained through meta-analyses. PIs show the extent of between-study variation and predict the possible effect in a future study that is comparable to those included in the meta-analysis. Single proportions were logit transformed to maintain symmetry in the random effects meta-analysis. Confidence intervals for individual studies were estimated with the Wilson score. Heterogeneity across the included studies was evaluated by visual inspection of forest plots, as estimates of *I*-squared are uninformative in meta-analyses of single proportions. Stratified random-effects meta-analyses were planned to evaluate the impact on mortality of COVID-19 severity at study entry (i.e., severe vs non-severe), older age (< 60 vs ≥ 60 years), presence of comorbidities, concomitant arterial hypertension, admission to intensive care unit (ICU), and requirement for invasive ventilation, if at least 10 studies contributed to the analyses. Stratified analyses were planned only for peer-reviewed studies. The incidence of all-cause mortality in patients developing ARDS was also evaluated in stratified analysis. Severe cases of COVID-19 develop complications like ARDS, shock, acute kidney injury, or cardiac injury more often during the first 2 weeks of hospitalization [9]. Thus, an additional stratified analysis was planned to explore the effects of follow-up duration on the primary outcomes comparing adequate follow-up duration versus inadequate follow-up duration [21].

Statistical analyses were performed using R studio version 1.2.5001, “meta” and “forestplot” packages [22].

## Results

A total of 4540 records were identified from the search of MEDLINE, EMBASE, and PubMed databases, and 1559 additional records were found in the medRxiv archive (Additional file 1: Fig. S1). After removing 1642 duplicates, 4354 records were excluded based on title and abstract screening. Of the remaining 103 records, 24 were excluded by full-text evaluation. Finally, a total of 44 peer-reviewed studies (one RCT and 43 observational studies) including 14,866 patients with laboratory-confirmed COVID-19 were considered in the primary analysis [1, 23–65], and 35 not peer-reviewed studies (one RCT and 34 observational studies) including 11,283 patients were evaluated in the secondary analysis (Additional file 1). The inter-reviewer agreement was excellent with a kappa statistic of 0.92.

### Characteristics of included studies

The main characteristics of peer-reviewed studies are reported in Additional file 1: Table S3. Forty-one studies were conducted in China or other Asian countries, 2 in Western Europe, and 1 involved centers from the USA, Canada, Europe, and Japan. Twenty-eight studies (63.6%) were single centre and 16 (36.4%) multicentre. The size of the study population ranged from 52 to 1591 patients. Forty-three studies (97.7%) enrolled only hospitalized patients, while one study considered both outpatients and inpatients. Median hospital stay was 15 days (range 8 to 29; 12 studies). All studies reported at least one of the review primary outcomes, and 15 (34.1%) provided data on one or more secondary outcomes.

The risk of bias of the peer-reviewed RCT was high for blinding of participants and personnel, unclear for blinding of outcome assessors, and low for all other domains. The methodological quality of the 43 observational studies, one ambispective and 42 retrospective cohorts, is summarized in Additional file 1: Fig. S2 and Table S4. Sixteen studies (37%) reported consecutive inclusion of patients. Follow-up duration was adequate in seven studies and reported to be at least 2 weeks in thirteen studies. The main characteristics of not peer-reviewed studies are shown in Additional file 1: Tables S5 and S6.

### Patient characteristics

The main patient characteristics reported in peer-reviewed studies are given in Additional file 1: Table S7. The mean age ranged between 38 and 69 years, and 8370 (56.4%) patients were males. The most common symptoms on admission were fever (7853/9822 patients [79.9%]; 32 studies), cough (5965/9487 patients [62.9%]; 30 studies), and dyspnea (2067/8810 patients [23.5%]; 25 studies). The most common comorbidities were hypertension (2409/10310 patients [23.3%]; 29 studies), diabetes (1116/10435 patients [10.7%]; 31 studies), cardiovascular disease (1018/10782 patients [9.4%]; 32 studies), and cancer (397/11019 patients [3.6%]; 29 studies). Pre-existent respiratory system disease was reported in 2.8% of patients (312/11033 patients; 30 studies). Co-infection was diagnosed in 14.0% of patients (255/1815 patients; 10 studies).

Twenty-nine studies (65.9%) reported the severity of COVID-19 at study entry according to the criteria of American Thoracic Society (3 studies), the National Health Commission of the People’s Republic of China (10 studies), the World Health Organization criteria (9 studies), or other classifications (7 studies). As many as 41.3% (5175/12530) of patients were considered to have severe COVID-19 at study entry.

Most patients were managed in general wards, and 34.6% (2659/7687; 18 studies) were admitted to ICU. Supplemental oxygen was administered to 36.3% of patients (1959/5392; 18 studies), non-invasive ventilation to 8.7% (589/6797; 20 studies), and invasive-mechanical ventilation to 18.5% (1644/8901; 21 studies; see Additional file 1: Table S8), whereas 0.9% of the patients required extracorporeal membrane oxygenation (39/4412; 16 studies). Continuous renal replacement therapy was used in 1.9% of patients (86/4601; 14 studies).

The main characteristics of patients included in not peer-reviewed studies are reported in Additional file 1: Table S9. Twenty-four not peer-reviewed studies (68.6%) reported the severity of COVID-19 according to the criteria of American Thoracic Society (2 studies), the National Health Commission of the People’s Republic of China (4 studies), the World Health Organization criteria (4 studies), or other classifications (14 studies). As many as 31.8% (1902/5975) of patients of not peer-reviewed studies were considered to have severe COVID-19 at study entry. The type of treatment provided in not peer-reviewed studies is shown in Additional file 1: Table S10.

### Clinical outcomes

**Fig. 1:**
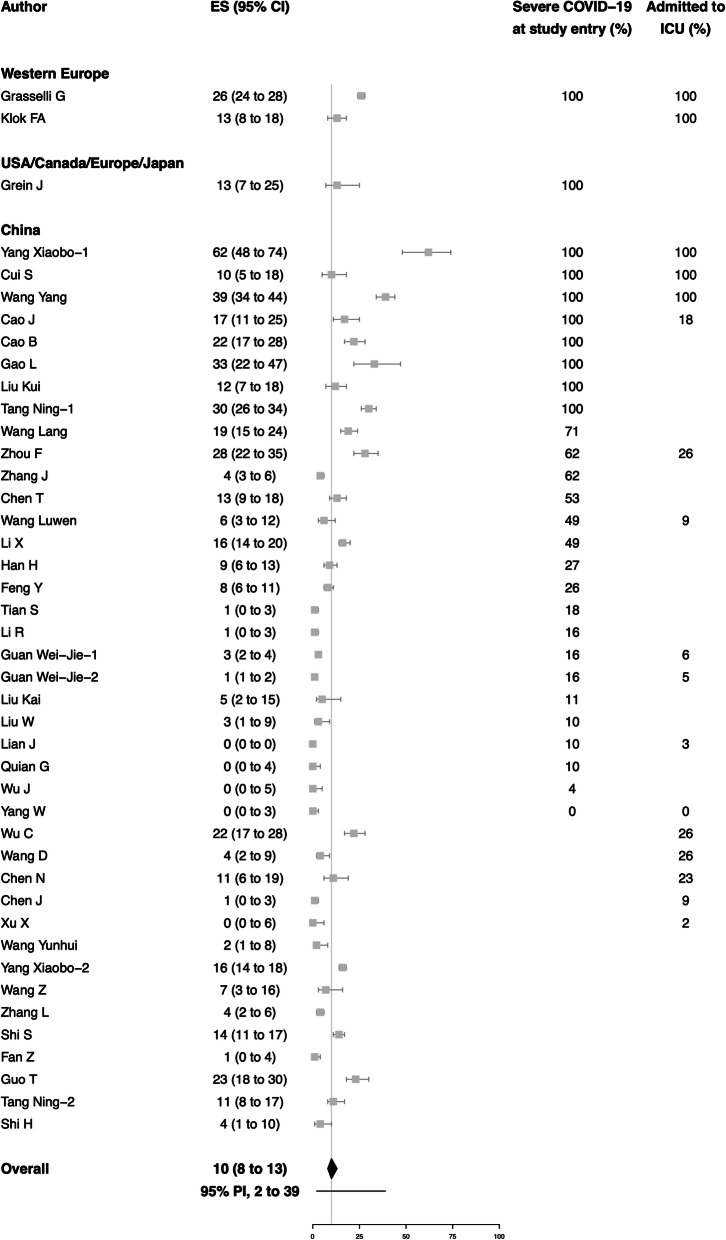
All-cause mortality in patients with COVID-19. All-cause mortality in peer-reviewed studies from Asian and Western countries, sorted by severity of COVID-19 at study entry and the proportion of patients admitted to ICU. The vertical line indicates the summary estimate. Gray squares indicate individual study estimates of the proportion of all-cause mortality, whereas the gray horizontal lines indicate 95% confidence intervals of the individual studies. The diamond indicates the summary estimate with 95% confidence intervals. The horizontal black line refers to the prediction intervals which are displayed numerically under the 95% confidence intervals. CI, confidence intervals; COVID-19, Coronavirus disease 2019; ES, estimates; ICU, intensive care unit; PI, prediction intervals; USA, United States of America

**Fig. 2:**
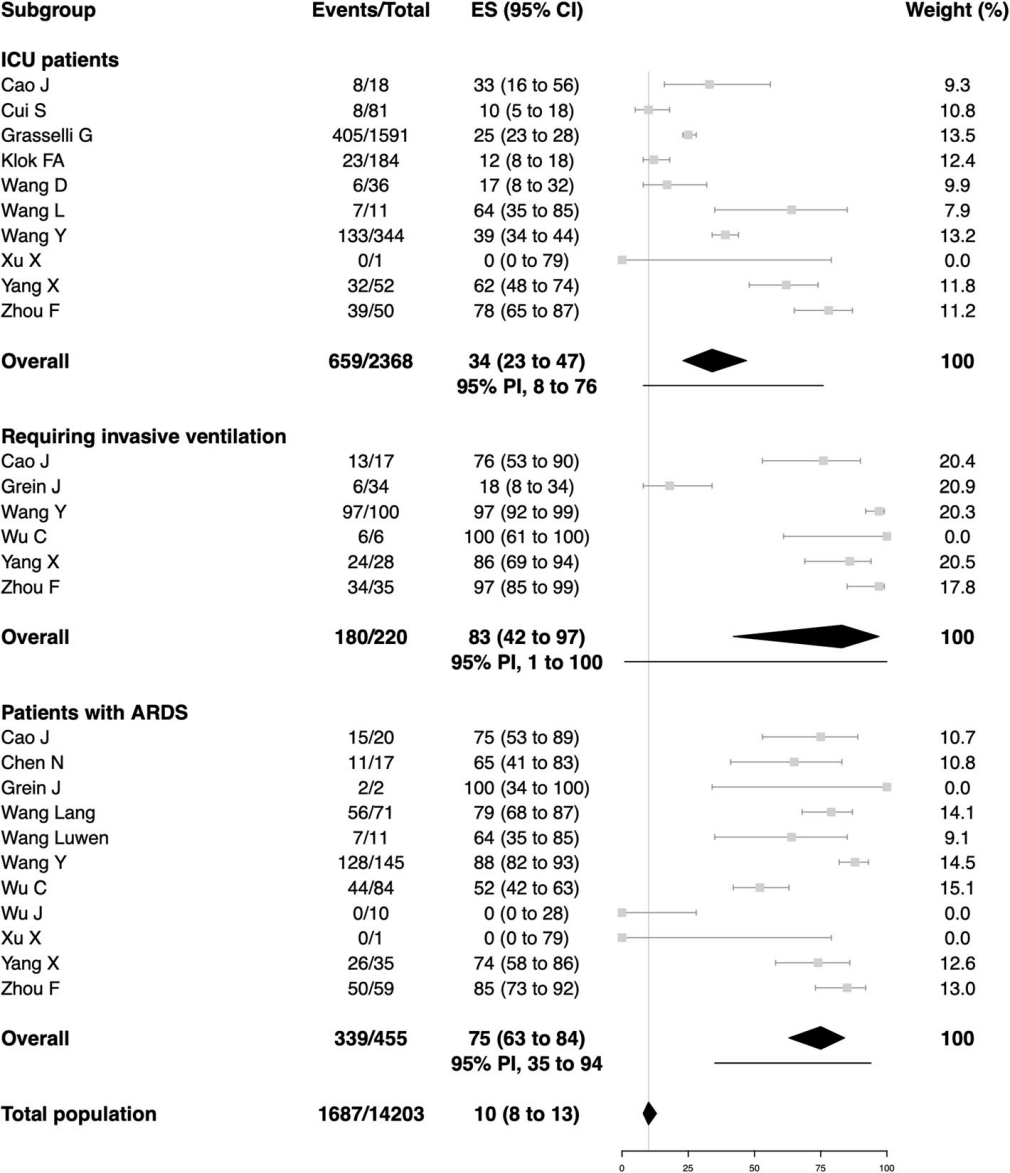
All-cause mortality in patients admitted to ICU, requiring invasive ventilation, and developing ARDS. The vertical line indicates the summary estimate for all-cause mortality in total population. Gray squares indicate individual study estimates of the proportion of all-cause mortality, whereas the gray horizontal lines indicate 95% confidence intervals of the individual studies. The diamonds indicate the summary estimates with 95% confidence intervals. The horizontal black lines refer to the prediction intervals which are displayed numerically under the 95% confidence intervals. ARDS, acute respiratory distress syndrome; CI, confidence intervals; COVID-19, Coronavirus disease 2019; ES, estimates; ICU, intensive care unit; PI, prediction intervals

Figure 1 shows all-cause mortality in peer-reviewed studies from Asian and Western countries, sorted by severity of COVID-19 at study entry and the proportion of patients admitted to ICU. The summary estimate for all-cause mortality was 10% with substantial between-study heterogeneity and large 95% PI (95% PI, 2 to 39%; 1687/14203 patients; 43 studies) suggesting high uncertainty. All-cause mortality was 34% in patients admitted to ICU (95% PI, 8 to 76%; 659/2368 patients; 10 studies), 83% in patients requiring invasive ventilation (95% PI, 1 to 100%; 180/220 patients; 6 studies), and 75% in patients who developed ARDS (95% PI, 35 to 94%; 339/455 patients; 11 studies) with very high between-study heterogeneity (Figure 2). All-cause mortality in patients with advanced age, one or more comorbidities, concomitant arterial hypertension, and severe COVID-19 at study entry and in studies with adequate follow-up is shown in Additional file 1: Fig. S3.

**Fig. 3:**
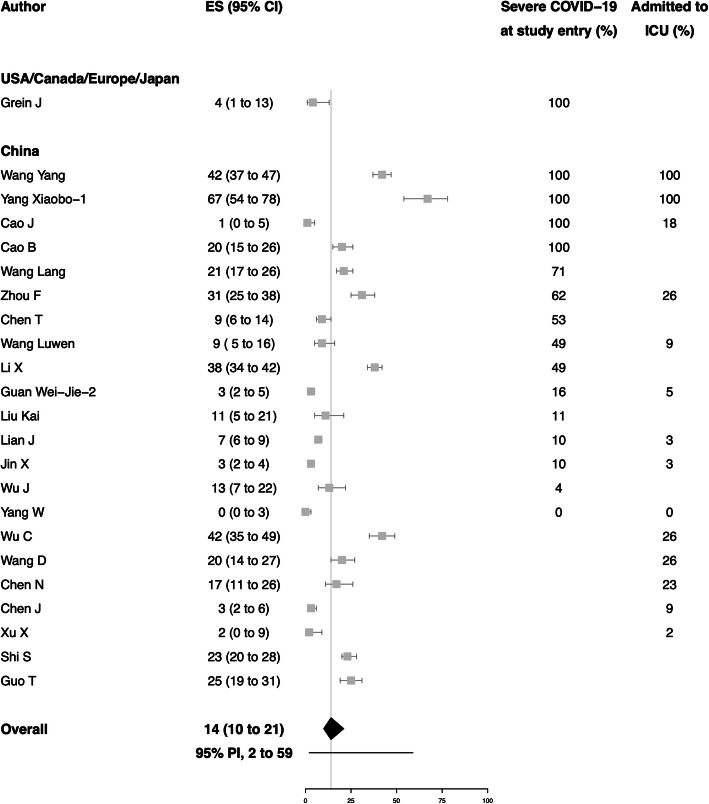
ARDS in patients with COVID-19. ARDS in peer-reviewed studies from Asian and Western countries, sorted by severity of COVID-19 at study entry and the proportion of patients admitted to ICU. The vertical line indicates the summary estimate. Gray squares indicate individual study estimates of the proportion of ARDS, whereas the gray horizontal lines indicate 95% confidence intervals of the individual studies. The diamond indicates the summary estimate with its 95% confidence intervals. The horizontal black line refers to the prediction intervals which are displayed numerically under the 95% confidence intervals. ARDS, acute respiratory distress syndrome; CI, confidence intervals; COVID-19, Coronavirus disease 2019; ES, estimates; ICU, intensive care unit; PI, prediction intervals; USA, United States of America

Figure 3 shows ARDS outcome data in peer-reviewed studies from Asian and Western countries sorted for severity of COVID-19 at study entry and the proportion of patients admitted to the ICU. On average, ARDS occurred in 14% of patients with significant between-study heterogeneity (95% PI, 2 to 59%; 999/6322 patients; 23 studies). Studies with follow-up until patient discharge or death reported significantly higher ARDS risks compared to studies with inadequate follow-up duration (28% vs 11%; *p* = 0.002; Additional file 1: Fig. S4).

**Fig. 4:**
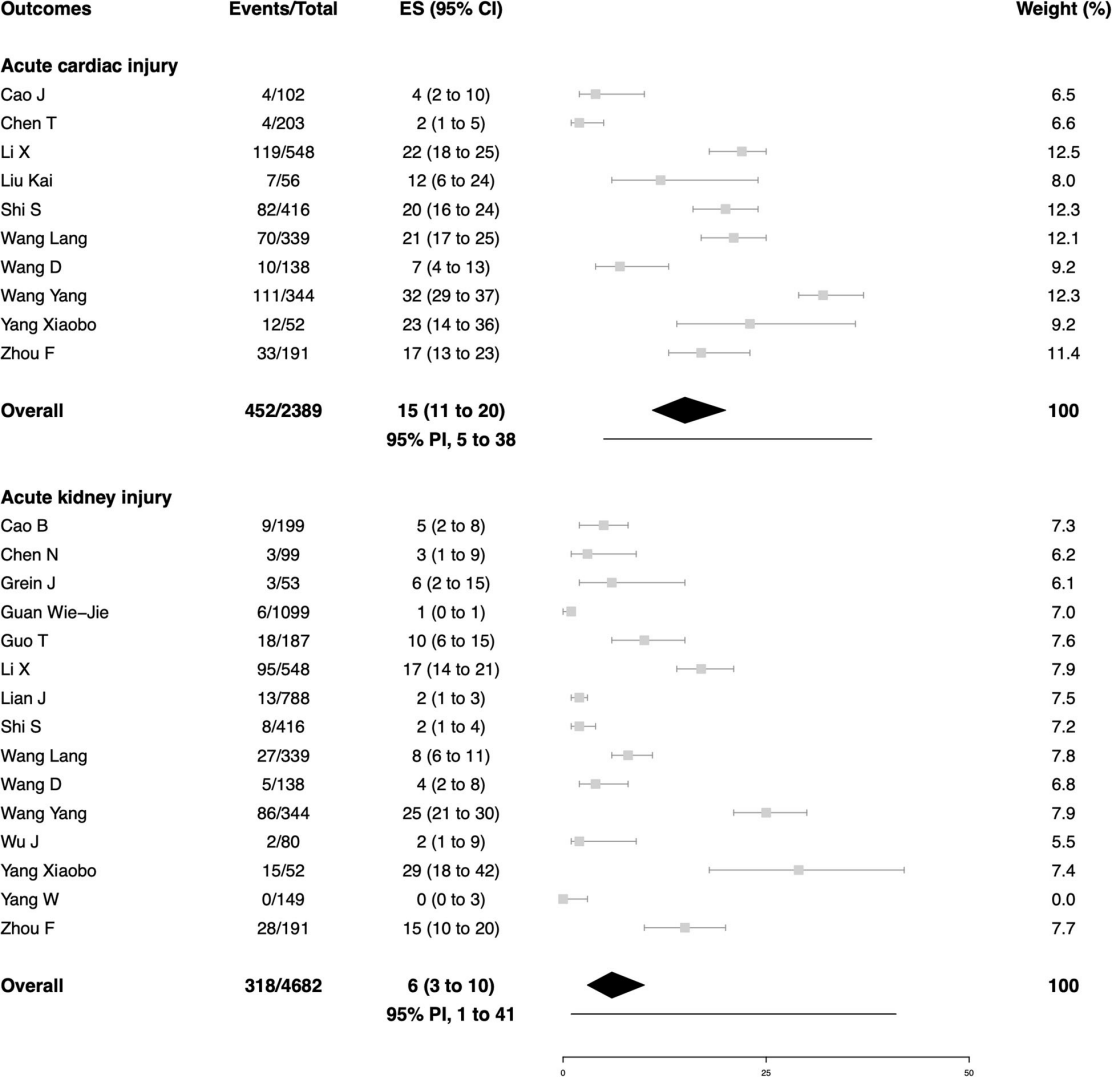
Acute cardiac and kidney injury in patients with COVID-19. Gray squares indicate individual study estimates of the proportion of the outcomes, whereas the gray horizontal lines indicate 95% confidence intervals of the individual studies. The diamonds indicate the summary estimates with 95% confidence intervals. The horizontal black lines refer to the prediction intervals which are displayed numerically under the 95% confidence intervals. CI, confidence intervals; COVID-19, Coronavirus disease 2019; ES, estimates; PI, prediction intervals

**Fig. 5:**
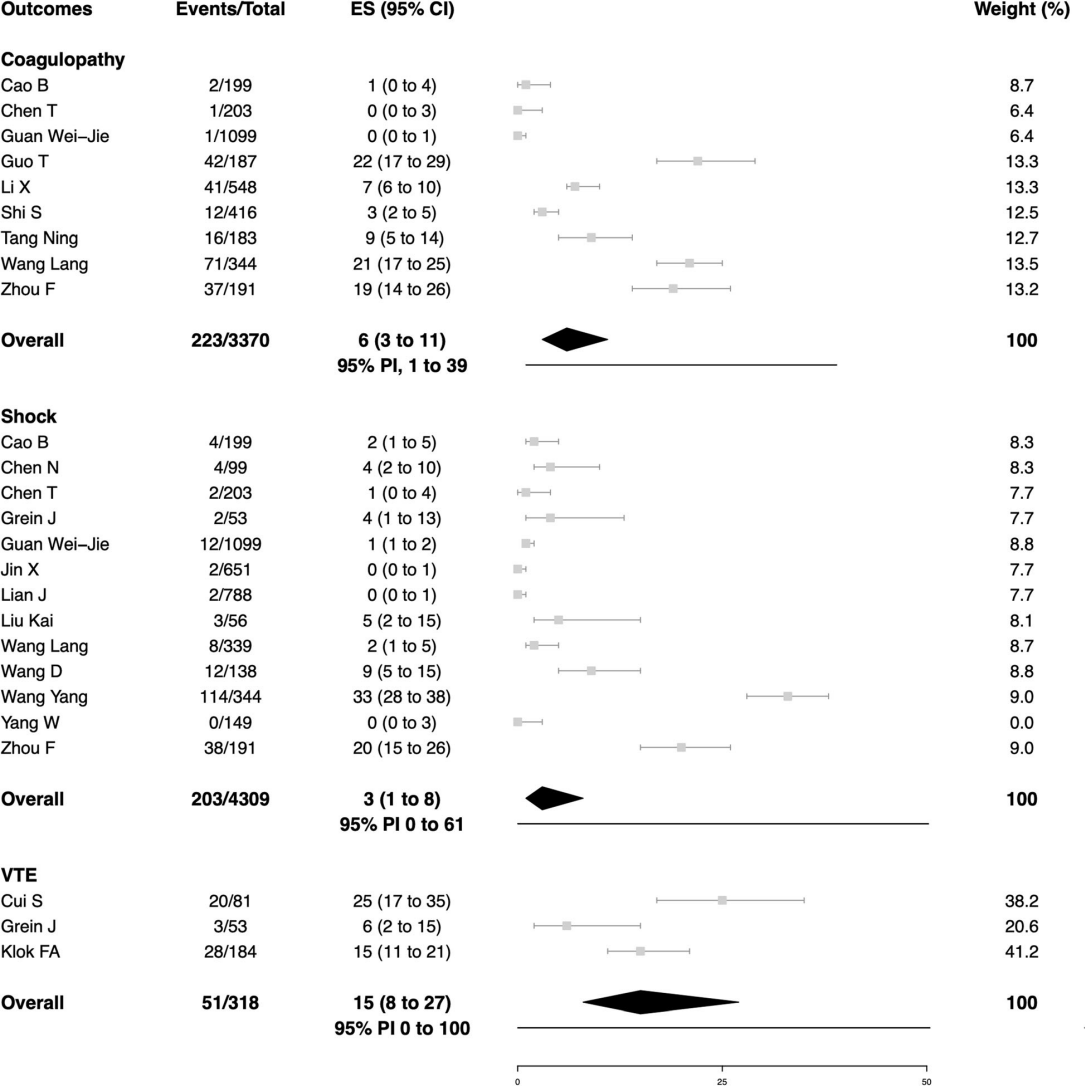
Coagulopathy, shock, and venous thromboembolism in patients with COVID-19. Gray squares indicate individual study estimates of the proportion of the outcomes, whereas the gray horizontal lines indicate 95% confidence intervals of the individual studies. The diamonds indicate the summary estimates with 95% confidence intervals. The horizontal black lines refer to the prediction intervals which are displayed numerically under the 95% confidence intervals. CI, confidence intervals; COVID-19, Coronavirus disease 2019; ES, estimates; PI, prediction intervals; VTE, venous thromboembolism

Figures 4 and 5 and Additional file 1: Table S11 show outcome data for the secondary outcomes. In peer-reviewed studies, the average summary estimates were 15% for acute cardiac injury (95% PI, 5 to 38%; 452/2389 patients; 10 studies), 15% for venous thromboembolism (95% PI, 0 to 100%; patients; 3 studies), 6% for acute kidney injury (95% PI, 1 to 41%; 318/4682 patients; 15 studies), 6% for coagulopathy (95% PI, 1 to 39%; 223/3370 patients; 9 studies), and 3% for shock (95% PI, 0 to 61%; 203/4309 patients; 13 studies). Major bleeding events were reported in one study (2/199 patients; 1.0%).

The incidence of the primary and secondary outcomes in not peer-reviewed studies was consistent with that of the primary analysis (Additional file 1: Table S12 and Figs. S5 to S10). As observed in peer-reviewed studies, all summary estimates for primary and secondary outcomes presented significant between-study heterogeneity and wide prediction intervals.

## Discussion

In this meta-analysis including 14,866 symptomatic adult patients with COVID-19, all-cause mortality was 10%. The incidence seemed three to eightfold higher in patients admitted to ICU, requiring invasive ventilation, and those who developed ARDS based on very low quality evidence due to heterogeneity and risk of bias.

On average, mortality of COVID-19 patients admitted to ICU was 34%, consistent with the results of a recent large study of ICU patients with infection of the respiratory tract or other sites [66]. In ICU patients as well as in those requiring invasive ventilation or developing ARDS, prediction intervals were wide indicating very low confidence in the summary estimates. Overall, these findings suggest that different case mix with different prognosis were included in the studies. Potential explanation for heterogeneity include differences in age and comorbidities between study populations, definition of COVID-19-related deaths, and susceptibility or response to COVID-19 [6, 67, 68]. Another potential explanation for the heterogeneous risks of mortality and ARDS may be related to the insufficient length of follow-up. The proportion of patients who were still hospitalized at the end of the study period was as high as 31.9% (Additional file 1: Tables S11). The clinical outcomes of these patients remain unknown, which may have underestimated the actual incidence of complications which typically tend to occur during the late hyperinflammatory phase of the disease. Only 7 studies followed patients until death or discharge, and 13 studies had a follow-up of at least 14 days, which may be regarded as a minimum follow-up duration to observe clinically relevant outcomes [21]. Stratified analysis suggested that mortality and ARDS were significantly higher in studies with adequate (20% and 28%) compared to those with inadequate follow-up duration (9% and 11%, respectively).

With regard to the secondary outcomes of this meta-analysis, acute cardiac injury and venous thromboembolism seemed to represent the most frequent complications during hospitalization, followed by acute kidney injury, coagulopathy, and shock. As observed for the primary outcomes, the uncertainty around all these estimates was large. Importantly, information on secondary outcomes was provided by only one third of the studies, suggesting significant underreporting. In addition, it is unclear if these outcomes were systematically evaluated in all patients, and we cannot exclude that our summary estimates may represent an underestimation of actual incidence. Nonetheless, our findings suggest that these acute complications are not infrequent and may have a relevant impact on patient prognosis and burden for health care systems. A better understanding of the risk and prompt recognition of these complications could help to improve policies for diagnosis and prevention, resource allocation, and help the design of future interventional studies.

The current review has some limitations which need to be discussed. Most studies were conducted in China or other Asian countries which contributed to 88.1% of all patients. The different accessibility and quality of health care settings and resources within each country and between countries may influence the representation of clinical outcomes and limit interpretation of some of the findings. Most studies were retrospective, and it was often unclear whether patients were enrolled consecutively. The need to provide objective findings and the urgent timeline of the disease may have affected the quality of data recording and reporting. The study-level analysis and poor reporting limited the possibility to evaluate the effects of specific patient characteristics, type of medical and supportive treatment (e.g., requirement for kidney replacement therapy) on clinical outcomes. All outcomes were provided as crude proportions rather than time-to-event rates (e.g., events per 100 patient days), which incorporate a measure of the at-risk period, thereby giving a more standardized and less biased summary estimate. Finally, in light of the large between-study heterogeneity observed, pooling of the individual study estimates is debatable. For this reason, we focused on the prediction interval rather than the confidence interval when describing results.

## Conclusions

This systematic review and meta-analysis summarized the current evidence on the incidence of mortality and acute complications in hospitalized patients with COVID-19. Mortality was very high in critically ill patients, based on very low-quality evidence with striking heterogeneity. Health care professionals caring for COVID-19 patients need to be aware of this variability for medical decision making and communication.

## Supplementary information

**Additional file 1: Table S1.** Search strategy, update in Embase and Medline. **Table S2.** Search strategy in Pubmed. **Table S3.** Characteristics of peer-reviewed studies. **Table S4.** MINORS quality assessment for peer-reviewed studies. **Table S5.** Characteristics of not peer-reviewed studies. **Table S6.** MINORS quality assessment for not peer-reviewed studies. **Table S7.** Patient demographics and clinical characteristics at study entry in peer-reviewed studies. **Table S8.** Treatment provided in peer-reviewed studies. **Table S9**. Patient demographics and clinical characteristics at study entry in not peer-reviewed studies. **Table S10.** Treatment provided in not peer-reviewed studies. **Table S11.** Clinical outcomes of patients in peer-reviewed studies. **Table S12.** Clinical outcomes of patients in not peer-reviewed studies. **Fig. S1.** PRISMA flow diagram. F**ig. S2.** MINORS quality assessment for peer reviewed (panel A) and not peer-reviewed (panel B) studies. **Fig. S3**. All-cause mortality by subgroup of patients with COVID-19, peer-reviewed studies. **Fig. S4.** ARDS by follow-up duration, peer-reviewed studies. **Fig. S5.** All-cause mortality in patients with COVID-19, not peer-reviewed studies. **Fig. S6**. ARDS in patients with COVID-19, not peer-reviewed studies. **Fig. S7**. Acute cardiac injury in patients with COVID-19, not peer-reviewed studies. **Fig. S8**. Acute kidney injury in patients with COVID-19, not peer-reviewed studies. **Fig. S9**. Coagulopathy in patients with COVID-19, not peer-reviewed studies. **Fig. S10**. Shock in patients with COVID-19, not peer-reviewed studies.

## Data Availability

The datasets used and/or analyzed during the current study are available from the corresponding author on reasonable request.

## Abbreviations

ARDS: Acute respiratory distress syndrome
CIs: Confidence intervals;
COVID-19: *Coronavirus* disease 2019
ICU: Intensive care unit
MINORS: Methodological index for non-randomized studies
PIs: Prediction intervals
PRISMA: Preferred Reporting Items for Systematic reviews and Meta-analysis
RCTs: Randomized controlled trials
