# Supplementary material for: Acute complications and mortality in hospitalized patients with coronavirus disease 2019: a systematic review and meta-analysis
Source: Crit Care. 2020 Jul 2;24:389. doi: 10.1186/s13054-020-03022-1 (PMC7330272; doi:10.1186/s13054-020-03022-1)
Supplement: Supplementary file 1 — Additional file 1: Table S1. Search strategy, update in Embase and Medline. Table S2. Search strategy in Pubmed. Table S3. Characteristics of peer-reviewed studies. Table S4. MINORS quality assessment for peer-reviewed studies. Table S5. Characteristics of not peer-reviewed studies. Table S6. MINORS quality assessment for not peer-reviewed studies. Table S7. Patient demographics and clinical characteristics at study entry in peer-reviewed studies. Table S8. Treatment provided in peer-reviewed studies. Table S9. Patient demographics and clinical characteristics at study entry in not peer-reviewed studies. Table S10. Treatment provided in not peer-reviewed studies. Table S11. Clinical outcomes of patients in peer-reviewed studies. Table S12. Clinical outcomes of patients in not peer-reviewed studies. Fig. S1. PRISMA flow diagram. Fig. S2. MINORS quality assessment for peer reviewed (panel A) and not peer-reviewed (panel B) studies. Fig. S3. All-cause mortality by subgroup of patients with COVID-19, peer-reviewed studies. Fig. S4. ARDS by follow-up duration, peer-reviewed studies. Fig. S5. All-cause mortality in patients with COVID-19, not peer-reviewed studies. Fig. S6. ARDS in patients with COVID-19, not peer-reviewed studies. Fig. S7. Acute cardiac injury in patients with COVID-19, not peer-reviewed studies. Fig. S8. Acute kidney injury in patients with COVID-19, not peer-reviewed studies. Fig. S9. Coagulopathy in patients with COVID-19, not peer-reviewed studies. Fig. S10. Shock in patients with COVID-19, not peer-reviewed studies. [file 13054_2020_3022_MOESM1_ESM.pdf]

Table S1. Search strategy, update in Embase and Medline

	EMBASE#	Results		**MEDLINE#**	Results
1	coronavirus.ti,ab.	11196	1	coronavirus.ti,ab.	10557
2	corona virus.ti,ab.	299	2	corona virus.ti,ab.	225
3	coronavirinae.ti,ab.	12	3	coronavirinae.ti,ab.	13
4	coronaviridae.ti,ab.	290	4	coronaviridae.ti,ab.	278
5	betacoronavirus.ti,ab.	184	5	betacoronavirus.ti,ab.	176
6	covid19.ti,ab.	0	6	covid19.ti,ab.	4
7	covid 19.ti,ab.	543	7	covid 19.mp.	1168
8	nCoV.ti,ab.	285	8	nCoV.ti,ab.	420
9	CoV2.ti,ab.	18	9	CoV2.ti,ab.	18
10	sars cov 2.ti,ab.	206	10	sars cov 2.ti,ab.	316
11	"cov 2".ti,ab.	223	11	"cov 2".ti,ab.	325
12	2019ncov.ti,ab.	1	12	2019ncov.ti,ab.	2
13	"novel cov".ti,ab.	13	13	"novel cov".ti,ab.	16
14	"wuhan virus".ti,ab. 3c20	2	14	"wuhan virus".ti,ab.	3
15	(wuhan or Hubei or huanan).ti,ab.	5590	15	(wuhan or Hubei or huanan).ti,ab.	4207
16	("severe acute respiratory" or pneumonia).ti,ab.	181945	16	("severe acute respiratory" or pneumonia).ti,ab.	117510
17	exp severe acute respiratory syndrome/	8222	17	exp severe acute respiratory syndrome/	4470
18	exp pneumonia/	314802	18	exp pneumonia/	90583
19	or/16-18	351943	19	or/16-18	159068
20	outbreak.ti,ab.	66170	20	outbreak.ti,ab.	55728
21	and/15,19-20	96	21	and/15,19-20	162
22	exp Coronavirinae/	11286	22	exp Coronavirinae/	12598
23	coronavirus infections.sh.	252	23	coronavirus infections.sh.	4493
24	covid-19.sh.	3	24	covid-19.sh.	0
25	severe acute respiratory syndrome coronavirus 2.sh.	19	25	severe acute respiratory syndrome coronavirus 2.sh.	0
26	exp Betacoronavirus/	6764	26	exp Betacoronavirus/	6432
27	or/1-14,21-26	17252	27	Severe Acute Respiratory Syndrome/	4470
28	limit 27 to yr="2020 -Current"	1453	28	exp Coronavirus/	11425

			29	exp Coronavirus Infections/	9723
			30	severe acute respiratory syndrome coronavirus 2.mp.	306
			31	exp Betacoronavirus/	6432
			32	or/1-14,21-31	19648
			33	limit 32 to ed=20191201-20200324	534
			34	remove duplicates from 33	521

#Searches run on March 24 2020 in Embase Classic and Embase and Ovid MEDLINE(R), retrieving citations from inception to March 23 2020, using the OVID SP platform.

	EMBASE§	Results		MEDLINE§	Results
**1**	coronavirus.ti,ab.	12384	1	coronavirus.ti,ab.	12222
**2**	corona virus.ti,ab.	361	2	corona virus.ti,ab.	304
**3**	coronavirinae.ti,ab.	12	3	coronavirinae.ti,ab.	13
**4**	coronaviridae.ti,ab.	296	4	coronaviridae.ti,ab.	295
**5**	betacoronavirus.ti,ab.	195	5	betacoronavirus.ti,ab.	198
**6**	covid19.ti,ab.	27	6	covid19.ti,ab.	42
**7**	covid 19.ti,ab.	2751	7	covid 19.mp.	4679
**8**	nCoV.ti,ab.	393	8	nCoV.ti,ab.	526
**9**	CoV2.ti,ab.	40	9	CoV2.ti,ab.	47
**10**	sars cov 2.ti,ab.	790	10	sars cov 2.ti,ab.	1163
**11**	"cov 2".ti,ab.	811	11	"cov 2".ti,ab.	1179
**12**	2019ncov.ti,ab.	2	12	2019ncov.ti,ab.	4
**13**	"novel cov".ti,ab.	13	13	"novel cov".ti,ab.	17
**14**	"wuhan virus".ti,ab.	3	14	"wuhan virus".ti,ab.	4
**15**	(wuhan or Hubei or huanan).ti,ab.	5975	15	(wuhan or Hubei or huanan).ti,ab.	4632
**16**	("severe acute respiratory" or pneumonia).ti,ab.	182983	16	("severe acute respiratory" or pneumonia).ti,ab.	118629
**17**	exp severe acute respiratory syndrome/	8361	17	exp severe acute respiratory syndrome/	4537
**18**	exp pneumonia/	316539	18	exp pneumonia/	91640
**19**	or/16-18	354060	19	or/16-18	160932
**20**	outbreak.ti,ab.	66837	20	outbreak.ti,ab.	56628
**21**	and/15,19-20	189	21	and/15,19-20	268
**22**	exp Coronavirinae/	12757	22	exp Coronavirinae/	13417

**23**	coronavirus infections.sh.	252	23	coronavirus infections.sh.	5392
**24**	covid-19.sh.	110	24	covid-19.sh.	0
**25**	severe acute respiratory syndrome coronavirus 2.sh.	392	25	severe acute respiratory syndrome coronavirus 2.sh.	0
**26**	exp Betacoronavirus/	7733	26	exp Betacoronavirus/	7224
**27**	or/1-14,21-26	20069	27	Severe Acute Respiratory Syndrome/	4537
**28**	limit 27 to yr="2020 -Current"	4247	28	exp Coronavirus/	12243
**29**	letter.pt.	1109811	29	exp Coronavirus Infections/	10626
**30**	editorial.pt.	649149	30	severe acute respiratory syndrome coronavirus 2.mp.	1340
**31**	conference abstract.pt.	3749649	31	exp Betacoronavirus/	7224
**32**	note.pt.	792782	32	or/1-14,21-31	23707
**33**	or/29-32	6301391	33	limit 32 to yr="2020 -Current"	5864
**34**	(exp animal/ or nonhuman/) not exp human/	7144518	34	editorial.pt.	524415
**35**	Hospitalization/	374011	35	letter.pt.	1071161
**36**	exp Mortality/	1121888	36	comment.pt.	841461
**37**	Critical Care/	97128	37	retracted-publication.pt.	7482
**38**	exp Intensive Care Units/	189013	38	clinical-conference.pt.	7330
**39**	exp Acute Kidney Injury/	87211	39	or/34-38	1844087
**40**	exp Disseminated Intravascular Coagulation/	25191	40	exp animals/ not humans.sh.	4691007
**41**	Shock, Cardiogenic/	14769	41	Hospitalization/	105236
**42**	Hemorrhage/	107048	42	exp Mortality/	376717
**43**	Respiratory Distress Syndrome, Adult/	17790	43	Critical Care/	51398
**44**	acute respiratory distress syndrom*.mp.	19783	44	exp Intensive Care Units/	82682
**45**	hospitali#ation*.ti,ab.	267487	45	exp Acute Kidney Injury/	45549
**46**	("intensive care unit$1" or icu$1).ti,ab.	223717	46	exp Disseminated Intravascular Coagulation/	10944
**47**	(acute adj5 kidney adj5 injur*).ti,ab.	35673	47	Shock, Cardiogenic/	8370
**48**	(acute adj5 cardiac adj5 injur*).ti,ab.	1995	48	Hemorrhage/	72178
**49**	(dvt$1 or vte$1 or "deep venous thromb*" or "pulmonary embol*" or "venous thromboembol*" or coagulopath*).ti,ab.	130340	49	Respiratory Distress Syndrome, Adult/	19095
**50**	Disseminated Intravascular Coagulation*.ti,ab.	13142	50	acute respiratory distress syndrom*.mp.	13082
**51**	major bleeding$1.ti,ab.	18672	51	hospitali#ation*.ti,ab.	154202
**52**	(clinical* adj5 relevant adj5 bleeding$1).ti,ab.	1538	52	("intensive care unit$1" or icu$1).ti,ab.	133934

**53**	(clinical* adj5 (outcome* or characteristic* or finding* or feature* or progression*)).mp.	1511572	53	(acute adj5 kidney adj5 injur*).ti,ab.	21470
**54**	critical care.ti,ab.	45356	54	(acute adj5 cardiac adj5 injur*).ti,ab.	1303
**55**	shock.ti,ab.	247235	55	(dvt$1 or vte$1 or "deep venous thromb*" or "pulmonary embol*" or "venous thromboembol*" or coagulopath*).ti,ab.	79037
**56**	case fatalit*.ti,ab.	12004	56	Disseminated Intravascular Coagulation*.ti,ab.	9726
**57**	"*Characteristics".kw.	16885	57	major bleeding$1.ti,ab.	9617
**58**	(death or died or mortality).ti,ab.	2188381	58	(clinical* adj5 relevant adj5 bleeding$1).ti,ab.	750
**59**	artificial ventilation/	137911	59	(clinical* adj5 (outcome* or characteristic* or finding* or feature* or progression*)).mp.	599849
**60**	(invasive adj5 ventilation).mp.	12970	60	critical care.ti,ab.	26678
**61**	exp hospital discharge/	122495	61	shock.ti,ab.	174125
**62**	(hospital adj5 discharge*).mp.	165220	62	case fatalit*.ti,ab.	9427
**63**	or/35-62	4783482	63	"*Characteristics".kw.	524
**64**	27 not 33	16479	64	(death or died or mortality).ti,ab.	1442393
**65**	64 not 34	8629	65	Respiration, Artificial/	48046
**66**	and/63,65	2368	66	(invasive adj5 ventilation).mp.	5926
**67**	limit 66 to yr="2020 -Current"	708	67	intubation$1.mp.	76264
**68**	remove duplicates from 67	699	68	or/41-67	2771114
**69**	limit 68 to english language	640	69	32 not 39	21003
			70	69 not 40	13531
			71	and/68,70	2768
			72	limit 71 to yr="2020 -Current"	948
			73	remove duplicates from 72	910
			74	limit 73 to english language	805

§Searches run on April 20 2020 in Embase Classic+Embase and Ovid MEDLINE(R) ALL, using the OVID SP platform. We applied three filters to reduces the numbers needed to screen: a filter excluding irrelevant publication types, a filter excluding animal studies and a filter to retrieve studies focusing on clinical outcomes. This search strategy was validated using the 21 published reports identified in the initial search as reference set. Both searches in Embase and MEDLINE identified each of the 21 citations from the reference set.

Table S2. Search strategy in Pubmed

Search	Pubmed Query#	Results
**#4**	(#1 not #2) and #3	1121 - final set
**#3**	Search (((((((((((((((((((((((((((hospitalisation*[Title/Abstract]) OR hospitalization*[Title/Abstract]) OR acute respiratory distress syndrom*) OR intensive care unit*[Title/Abstract]) OR icus[Title/Abstract]) OR icu[Title/Abstract]) OR (acute and kidney and injur*[Title/Abstract])) OR (acute and cardiac and injur*[Title/Abstract])) OR dvt[Title/Abstract]) OR dvts[Title/Abstract]) OR vte[Title/Abstract]) OR vtes[Title/Abstract]) OR pulmonary embol*[Title/Abstract]) OR deep venous thromb*[Title/Abstract]) OR venous thromboembol*[Title/Abstract]) OR coagulopath*[Title/Abstract]) OR Disseminated Intravascular Coagulation*[Title/Abstract]) OR major bleeding*[Title/Abstract]) OR (clinical* and relevant and bleeding*[Title/Abstract])) OR (((outcome* or characteristic* or finding* or feature* or progression*) and clinical))) OR critical care[Title/Abstract]) OR shock[Title/Abstract]) OR case fatal*[Title/Abstract]) OR death[Title/Abstract]) OR died[Title/Abstract]) OR mortality[Title/Abstract]) OR (invasive and ventilation[Title/Abstract])) OR intubation[Title/Abstract]) OR mechanical ventilation[Title/Abstract]) OR (hospital and discharge*[Title/Abstract])	3607732
**#2**	Search ((((("letter"[Publication Type]) OR "comment"[Publication Type]) OR "editorial"[Publication Type]) OR (("retraction of publication"[Publication Type] OR retracted publication[Publication Type]))) OR "clinical conference"[Publication Type]) OR "congress"[Publication Type]	1928332
**#1**	Search (((coronavirus OR "corona virus" OR coronavirinae OR coronaviridae OR betacoronavirus OR covid19 OR "covid 19" OR nCoV OR "CoV 2" OR CoV2 OR sarscov2 OR 2019nCoV OR "novel CoV" OR "wuhan virus") OR ((wuhan OR hubei OR huanan) AND ("severe acute respiratory" OR pneumonia) AND (outbreak)) OR "Coronavirus"[Mesh] OR "Coronavirus Infections"[Mesh] OR "COVID-19" [Supplementary Concept] OR "severe acute respiratory syndrome coronavirus 2" [Supplementary Concept] OR "Betacoronavirus"[Mesh]) NOT medline [sb])	5768

#Searches run on April 20 2020, in pubmed.gov, advanced search builder. Search is set to capture citations not or not yet indexed in OVID MEDLINE.

Table S3. Characteristics of peer-reviewed studies

Study	Study design	Centers	Location	Setting	Inclusion, start date	Inclusion, end date
Cao B(1)	Open-label RCT	Single center	Wuhan, China	Hospitalized	18-Jan-20	3-Feb-20
Cao J(2)	Retrospective	Single center	Wuhan, China	Hospitalized	03-Jan-20	1-Feb-20
Chen J(3)	Retrospective	Single center	Shanghai, China	Hospitalized	20-Jan-20	6-Feb-20
Chen N(4)	Retrospective	Single center	Wuhan, China	Hospitalized	1-Jan-20	25-Jan-20
Chen T(5)	Retrospective	Single center	Wuhan, China	Hospitalized	01-Jan-20	10-Feb-20
Cui S(6)	Retrospective	Single center	China	Hospitalized	30-Jan-20	22-Feb-20
Fan Z(7)	Retrospective	Single center	Shangai, China	Hospitalized	20-Jan-20	29-Feb-20
Feng Y(8)	Retrospective	Multicenter	Wuhan, China	Hospitalized	01-Jan-20	15-Feb-20
Gao L(9)	Retrospective	Single center	Wuhan, China	Hospitalized	-	-
Grasselli G(10)	Retrospective	Multicenter	Lombardy,Italy	Hospitalized	20-Feb-20	18-Mar-20
Grein J(11)	Retrospective	Multicenter	US, Canada, Europe, Japan	Hospitalized	25-Jan-20	7-Mar-20
Guan Wei-jie(12)	Retrospective	Multicenter	China	Hospitalized	11-Dec-19	31-Jan-20
Guan Wei-jie(13)	Retrospective	Multicenter	China	Mixed	11-Dec-19	29-Jan-20
Guo T(14)	Retrospective	Single center	Wuhan, China	Hospitalized	23-Jan-20	23-Feb-20
Han H(15)	Retrospective	Single center	Wuhan, China	Hospitalized	01-Jan-20	18-Feb-20
Jin X(16)	Retrospective	Multicenter	Zhejiang province	Hospitalized	17-Jan-20	8-Feb-20
Klok FA(17)	Retrospective	Multicenter	Netherlands	Hospitalized	7-Mar-20	5-Apr-20
Li R(18)	Retrospective	Single center	China	Hospitalized	20-Jan-20	14-Feb-20
Li X(19)	Ambispective	Single center	Wuhan, China	Hospitalized	26-Jan-20	5-Feb-20
Lian J(20)	Retrospective	not specified	Zhejiang Province, China	Hospitalized	17-Jan-20	12-Feb-20
Liu Kay(21)	Retrospective	Single center	Hainan, China	Hospitalized	15-Jan-20	18-Feb-20
Liu Kui(22)	Retrospective	Multicenter	Hubei Province, China	Hospitalized	30-Dec-19	24-Jan-20

Liu W(23)	Retrospective	Multicenter	Wuhan, China	Hospitalized	30-Dec-19	15-Jan-20
Qian G(24)	Retrospective	Multicenter	Zhejiang Province, China	Hospitalized	20-Jan-20	11-Feb-20
Shi H(25)	Retrospective	Multicenter	Wuhan, China	Hospitalized	20-Dec-19	23-Jan-20
Shi S(26)	Retrospective	Single center	Wuhan, China	Hospitalized	20-Jan-20	10-Feb-20
Tang Ning(27)	Retrospective	Single center	Tongji, China	Hospitalized	1-Jan-20	13-Feb-20
Tang Ning(28)	Retrospective	Single center	Whuan, China	Hospitalized	1-Jan-20	3-Feb-20
Tian S(29)	Retrospective	Multicenter	Beijing, China	Hospitalized	20-Jan-20	10-Feb-20
Wang D(30)	Retrospective	Single center	Wuhan, China	Hospitalized	1-Jan-20	28-Jan-20
Wang Lang(31)	Retrospective	Single center	Wuhan, China	Hospitalized	1-Jan-20	6-Feb-20
Wang Luwen(32)	Retrospective	Single center	Wuhan, China	Hospitalized	14-Jan-20	13-Feb-20
Wang Yang(33)	Retrospective	Single center	Tongji, China	Hospitalized	25-Jan-20	25-Feb-20
Wang Yunhui(34)	Retrospective	Single center	Wuhan, China	Hospitalized	16-Jan-20	17-Feb-20
Wang Z(35)	Retrospective	Single center	Wuhan , China	Hospitalized	16-Jan-20	29-Jan-20
Wu C(36)	Retrospective	Single center	Wuhan, China	Hospitalized	25-Dec-19	26-Jan-20
Wu J(37)	Retrospective	Multicenter	Jiangsu Province, China	Hospitalized	22-Jan-20	14-Feb-20
Xu XW(38)	Retrospective	Multicenter	Zhejiang Province, China	Hospitalized	10-Jan-20	26-Jan-20
Yang W(39)	Retrospective	Multicenter	Wenzhou, China	Hospitalized	17-Jan-20	10-Feb-20
Yang Xiaobo(40)	Retrospective	Single center	Wuhuan, China	Hospitalized	24-Dec-19	26-Jan-20
Yang Xiaobo(41)	Retrospective	Single center	Wuhan, China	Hospitalized	late December	25-Feb-20
Zhang J(42)	Retrospective	Single center	Wuhan, China	Hospitalized	11-Jan-20	6-Feb-20
Zhang L(43)	Retrospective	Single center	Wuhan, China	Hospitalized	12-Jan-20	15-Mar-20
Zhou F(44)	Retrospective	Multicenter	Wuhan, China	Hospitalized	29-Dec-19	31-Jan-20

Table S4. MINORS quality assessment for peer-reviewed studies

Study	Clearly stated aim	Inclusion of consecutive patients	Prospective collection of data	Endpoints appropriate to the aim of the study	Unbiased assessment of the study endpoint	Follow-up period appropriate to the aim of the study	Loss to follow-up less than 5%	Prospective calculation of the study size
Cao J(2)	Adequate	Unclear	Inadequate	Death: Adequate<br>ARDS: Inadequate	ARDS: Unclear	Inadequate*	Adequate	Unclear
Chen J(3)	Adequate	Unclear	Inadequate	Death: Adequate<br>ARDS: Inadequate	ARDS: Unclear	Inadequate	Adequate	Unclear
Chen N(4)	Adequate	Adequate	Inadequate	Death: Adequate<br>ARDS: Inadequate	ARDS: Unclear	Inadequate	Adequate	Unclear
Chen T(5)	Adequate	Unclear	Inadequate	Death: Adequate<br>ARDS: Adequate	ARDS: Unclear	Inadequate	Adequate	Unclear
Cui S(6)	Adequate	Unclear	Inadequate	Death: Adequate	-	Inadequate	Adequate	Unclear
Fan Z(7)	Adequate	Adequate	Inadequate	Death: Adequate	-	Inadequate*	Unclear	Unclear
Feng Y(8)	Adequate	Unclear	Inadequate	Death: Adequate	-	Inadequate*	Adequate	Unclear
Gao L(9)	Adequate	Inadequate	Inadequate	Death: Adequate	-	Adequate	Adequate	Unclear
Grasselli G(10)	Adequate	Adequate	Inadequate	Death: Adequate	-	Inadequate	Adequate	Unclear
Grein J(11)	Adequate	Unclear	Inadequate	Death: Adequate<br>ARDS: Inadequate	-	Adequate	Adequate	Inadequate
Guan Wei-jie(12)	Adequate	Inadequate	Inadequate	Death: Adequate<br>ARDS: Unclear	ARDS: Unclear	Inadequate	Adequate	Unclear
Guan Wei-jie(13)	Adequate	Inadequate	Inadequate	Death: Adequate<br>ARDS: Adequate	ARDS: Unclear	Inadequate	Adequate	Unclear
Guo T(14)	Adequate	Inadequate	Inadequate	Death: Adequate	-	Adequate	Adequate	Unclear
Han H(15)	Adequate	Unclear	Inadequate	Death: Adequate	-	Inadequate*	Adequate	Unclear
Jin X(16)	Adequate	Unclear	Inadequate	Death: Adequate<br>ARDS: Adequate	ARDS: Unclear	Inadequate	Adequate	Unclear
Klok FA(17)	Adequate	Unclear	Inadequate	Death: Adequate	-	Inadequate	Adequate	Unclear
Li R(18)	Adequate	Unclear	Inadequate	Death: Adequate	-	Inadequate*	Adequate	Unclear
Li X(19)	Adequate	Adequate	Inadequate	Death: Adequate<br>ARDS: Inadequate	ARDS: Unclear	Adequate	Adequate	Inadequate
Lian J(20)	Adequate	Unclear	Inadequate	Death: Adequate<br>ARDS: Inadequate	ARDS: Unclear	Inadequate	Unclear	Unclear
Liu Kay(21)	Adequate	Unclear	Inadequate	Death: Adequate<br>ARDS: Inadequate	ARDS: Unclear	Unclear	Adequate	Unclear

Liu Kui(22)	Adequate	Unclear	Inadequate	Death: Adequate	-	Inadequate	Adequate	Unclear
Liu W(23)	Adequate	Unclear	Inadequate	Death: Adequate<br>ARDS: Adequate	ARDS: Unclear	Inadequate*	Unclear	Unclear
Qian G(24)	Adequate	Unclear	Inadequate	Death: Adequate<br>ARDS: Inadequate	ARDS: Unclear	Inadequate	Unclear	Unclear
Shi H(25)	Adequate	Inadequate	Inadequate	Death: Adequate<br>ARDS: Inadequate	ARDS: Unclear	Inadequate*	Adequate	Unclear
Shi S(26)	Adequate	Adequate	Inadequate	Death: Adequate<br>ARDS: Adequate	ARDS: Unclear	Inadequate	Adequate	Unclear
Tang Ning(27)	Adequate	Adequate	Inadequate	Death: Adequate	-	Inadequate*	Adequate	Unclear
Tang Ning(28)	Adequate	Unclear	Inadequate	Death: Adequate	-	Inadequate	Unclear	Unclear
Tian S(29)	Adequate	Unclear	Inadequate	Death: Adequate	-	Inadequate	Unclear	Unclear
Wang D(30)	Adequate	Adequate	Inadequate	Death: Adequate<br>ARDS: Adequate	ARDS: Unclear	Inadequate	Adequate	Unclear
Wang Lang(31)	Adequate	Adequate	Inadequate	Death: Adequate<br>ARDS: Adequate	ARDS: Unclear	Inadequate*	Adequate	Unclear
Wang Luwen(32)	Adequate	Unclear	Inadequate	Death: Adequate<br>ARDS: Inadequate	ARDS: Unclear	Inadequate	Adequate	Unclear
Wang Yang(33)	Adequate	Unclear	Inadequate	Death: Adequate<br>ARDS: Adequate	ARDS: Unclear	Adequate	Adequate	Unclear
Wang Yuhui(34)	Adequate	Unclear	Adequate	Death: Adequate	-	Inadequate	Adequate	Unclear
Wang Z(35)	Adequate	Adequate	Inadequate	Death: Adequate	-	Inadequate	Adequate	Unclear
Wu C(36)	Adequate	Unclear	Inadequate	Death: Adequate<br>ARDS: Adequate	ARDS: Unclear	Inadequate*	Adequate	Unclear
Wu J(37)	Adequate	Adequate	Inadequate	Death: Adequate<br>ARDS: Inadequate	ARDS: Unclear	Inadequate	Unclear	Unclear
Xu XW(38)	Adequate	Unclear	Inadequate	Death: Adequate<br>ARDS: Inadequate	ARDS: Unclear	Inadequate	Unclear	Unclear
Yang W(39)	Adequate	Adequate	Inadequate	Death: Adequate<br>ARDS: Inadequate	ARDS: Unclear	Inadequate	Adequate	Unclear
Yang Xiaobo(40)	Adequate	Unclear	Inadequate	Death: Adequate<br>ARDS: Adequate	ARDS: Unclear	Inadequate*	Adequate	Inadequate
Yang Xiaobo(41)	Adequate	Adequate	Inadequate	Death: Adequate	-	Adequate	Adequate	Unclear
Zhang J(42)	Adequate	Adequate	Inadequate	Death: Adequate<br>ARDS: Adequate	ARDS: Unclear	Unclear	Adequate	Unclear

Zhang L(43)	Adequate	Adequate	Inadequate	Death: Adequate<br>ARDS: Adequate	ARDS: Unclear	Unclear	Adequate	Unclear
Zhou F(44)	Adequate	Adequate	Inadequate	Death: Adequate<br>ARDS: Adequate	ARDS: Unclear	Adequate	Adequate	Unclear

*Patients followed-up for at least 2 weeks, until discharge or death

Table S5. Characteristics of not peer-reviewed studies

Study	Study design	Centers	Location	Setting	Inclusion, start date	Inclusion, end date
Benelli G(45)	Prospective	Single center	Crema, Italy	Hospitalized	21-Feb-20	19-Mar-20
Chen C(46)	RCT	Multicenter	Wuhan and Hubei Province, China	Hospitalized	20-Feb-20	12-Mar-20
Chen Xiaoping(47)	Retrospective	Single center	Wuhan, China	Hospitalized	5-Jan-20	7-Mar-20
Chen Xu(48)	Retrospective	Multicenter	Hunan Province, China	Hospitalized	23-Jan-20	14-Feb-20
Cheng Y(49)	Retrospective	Single center	Wuhan, China	Hospitalized	28-Jan-20	11-Feb-20
Cummings MJ(50)	Prospective	Multicenter	New York, USA	Hospitalized	2-Mar-20	1-Apr-20
Fan L(51)	Retrospective	Single center	Shenyang, China	Hospitalized	20-Jan-20	15-Mar-20
Feng Z(52)	Retrospective	Multicenter	South China, China	Hospitalized	17-Jan-20	28-Feb-20
Fu L(53)	Retrospective	Single center	Wuhan, China	Hospitalized	1-Jan-20	30-Jan-20
Giorgi Rossi P(54)	Prospective	Multicenter	Reggio Emilia, Italy	Mixed	27-Feb-20	2-Apr-20
Hu L(55)	Retrospective	Single center	Wuhan, China	Hospitalized	8-Jan-02	20-Feb-20
Jiang X(56)	Retrospective	Single center	Wuxi, China	Hospitalized	23-Jan-20	16-Feb-20
Joharatnam-Hogan N(57)	Retrospective	Multicenter	London, England	Hospitalized	12-Mar-20	7-Apr-20
Liu J(58)	Retrospective	Single center	Wuhan, China	Hospitalized	16-Jan-20	15-Feb-20
Liu Qibin(59)	Retrospective	Multicenter	Tongji, Cjina	Hospitalized	13-Dec-19	21-mar-20
Liu Qibin(60)	Retrospective	Single center	Tongji, China	Hospitalized	31-Jan-20	8-Mar-20
Liu Yanli(61)	Retrospective	Single center	Wuhan, China	Hospitalized	2-Jan-20	1-Feb-20
Liu Youbin(62)	Retrospective	Single center	Guangzhou, China	Hospitalized	10-Jan-20	24-Feb-20
Lu X(63)	Retrospective	Single center	Tongji, China	Hospitalized	25-Jan-20	25-Feb-20
Magagnoli J(64)	Retrospective	Multicenter	South Carolina, USA	Hospitalized	9-mar-20	11-Apr-20
Qi D(65)	Retrospective	Multicenter	Chongqing, China	Hospitalized	19-Jan-20	16-Feb-20
Qiu C(66)	Retrospective	Multicenter	Hunan Province, China	Hospitalized	22-Jan-20	12-Feb-20
Rubin SJ(67)	Retrospective	Single center	California, USA	Mixed	16-mar-20	-
Wang Z(68)	Retrospective	Multicenter	Sichuan Province, China	Hospitalized	16-Jan-20	3-Apr-20
Wen Y(69)	Retrospective	-	Shenzhen, China	Hospitalized	1-Jan-20	28-Feb-20
Wu C(70)	Retrospective	Single center	Wuhan, China	Hospitalized	25-Oct-20	27-Jan-20
Wu Y(71)	Retrospective	Single center	Wuhan, China	Hospitalized	-	2-Feb-20
Xiao G(72)	Retrospective	Single center	Wuhan, China	Hospitalized	5-Jan-2020	8-Mar-20
Xu H(73)	Retrospective	-	China	Hospitalized	2-Jan-20	14-Feb-20

Xu S(74)	Retrospective	Multicenter	Wuhan and Fuyang, China	Hospitalized	-	-
Xu Y(75)	Retrospective	Multicenter	China	Hospitalized	7-Feb-20	28-Feb-20
Yan S(76)	Retrospective	Multicenter	Hainan Province, China	Hospitalized	22-Jan-20	13-Mar-20
Zeng L(77)	Retrospective	Single center	Shenzhen, China	Hospitalized	1-Jan-20	29-Feb-20
Zhang G(78)	Retrospective	Single center	Wuhan, China	Hospitalized	2-Jan-20	10-Feb-20
Zhao W(79)	Retrospective	Single center	Beijing, China	Hospitalized	21-Jan-20	8-Feb-20

Table S6. MINORS quality assessment for not peer-reviewed studies

Study	Clearly stated aim	Inclusion of consecutive patients	Prospective collection of data	Endpoints appropriate to the aim of the study	Unbiased assessment of the study endpoint	Follow-up period appropriate to the aim of the study	Loss to follow-up less than 5%	Prospective calculation of the study size
Benelli G(45)	Adequate	Unclear	Adequate	Death: Adequate	-	Inadequate	Unclear	Unclear
Chen Xiaoping(47)	Adequate	Unclear	Inadequate	Death: Adequate	-	Inadequate*	Adequate	Unclear
Chen Xu(48)	Adequate	Adequate	Inadequate	Death: Adequate	-	Inadequate	Adequate	Unclear
Cheng Y(49)	Adequate	Adequate	Inadequate	Death: Adequate	-	Inadequate	Adequate	Unclear
Cummings MJ(50)	Adequate	Unclear	Adequate	Death: Adequate	-	Inadequate*	Adequate	Unclear
Fan L(51)	Adequate	Adequate	Inadequate	Death: Adequate<br>ARDS: Inadequate	ARDS: Unclear	Unclear	Adequate	Unclear
Feng Z(52)	Adequate	Unclear	Inadequate	Death: Adequate	-	Inadequate*	Adequate	Unclear
Fu L(53)	Adequate	Unclear	Adequate	Death: Adequate	-	Adequate	Adequate	Unclear
Giorgi Rossi P(54)	Adequate	Unclear	Inadequate	Death: Adequate	-	Inadequate	Adequate	Unclear
Hu L(55)	Adequate	Unclear	Inadequate	Death: Adequate<br>ARDS: Inadequate	ARDS: Unclear	Inadequate*	Adequate	Unclear
Jiang X(56)	Adequate	Unclear	Inadequate	Death: Adequate<br>ARDS: Adequate	ARDS: Unclear	Adequate	Adequate	Unclear
Joharatnam-Hogan N(57)	Adequate	Adequate	Inadequate	Death: Adequate	-	Unclear	Adequate	Unclear
Liu J(58)	Adequate	Unclear	Inadequate	Death: Adequate<br>ARDS: Inadequate	ARDS: Unclear	Inadequate	Adequate	Unclear
Liu Qibin(59)	Adequate	Inadequate	Inadequate	Death: Adequate	-	Adequate	Unclear	Unclear
Liu Qibin(60)	Adequate	Inadequate	Inadequate	Death: Adequate	-	Unclear	Adequate	Unclear
Liu Yanli(61)	Adequate	Unclear	Inadequate	Death: Adequate<br>ARDS: Adequate	ARDS: Unclear	Inadequate	Adequate	Unclear
Liu Youbin(62)	Adequate	Unclear	Inadequate	Death: Adequate<br>ARDS: Adequate	ARDS: Unclear	Unclear	Adequate	Unclear
Lu X(63)	Adequate	Unclear	Inadequate	Death: Adequate	-	Unclear	Adequate	Unclear
Magagnoli J(64)	Adequate	Inadequate	Inadequate	Death: Adequate	-	Adequate	Adequate	Unclear
Qi D(65)	Adequate	Inadequate	Inadequate	Death: Adequate<br>ARDS: Adequate	ARDS: Unclear	Inadequate	Adequate	Unclear
Qiu C(66)	Adequate	Unclear	Inadequate	Death: Adequate<br>ARDS: Adequate	ARDS: Unclear	Inadequate	Adequate	Unclear
Rubin SJ(67)	Adequate	Unclear	Inadequate	Death: Adequate<br>ARDS: Inadequate	ARDS: Unclear	Unclear	Adequate	Unclear
Wang Z(68)	Adequate	Unclear	Inadequate	Death: Adequate	-	Unclear	Adequate	Unclear
Wen Y(69)	Adequate	Unclear	Inadequate	Death: Adequate	-	Inadequate	Adequate	Unclear
Wu C(70)	Adequate	Inadequate	Inadequate	Death: Adequate<br>ARDS: Inadequate	ARDS: Unclear	Adequate	Adequate	Unclear
Wu Y(71)	Adequate	Unclear	Inadequate	Death: Adequate	-	Inadequate	Adequate	Unclear
Xiao G(72)	Adequate	Unclear	Inadequate	Death: Adequate	-	Unclear	Adequate	Unclear
Xu H(73)	Adequate	Adequate	Inadequate	Death: Adequate<br>ARDS: Adequate	ARDS: Unclear	Unclear	Adequate	Unclear
Xu S(74)	Adequate	Unclear	Inadequate	Death: Adequate	-	Unclear	Inadequate	Unclear

Xu Y(75)	Adequate	Adequate	Inadequate	Death: Adequate<br>ARDS: Inadequate	ARDS: Unclear	Inadequate	Adequate	Unclear
Yan S(76)	Adequate	Unclear	Inadequate	Death: Adequate<br>ARDS: Adequate	ARDS: Unclear	Adequate	Adequate	Unclear
Zeng L(77)	Adequate	Unclear	Inadequate	Death: Adequate	-	Inadequate	Adequate	Unclear
Zhang G(78)	Adequate	Unclear	Inadequate	Death: Adequate<br>ARDS: Adequate	ARDS: Unclear	Inadequate	Adequate	Unclear
Zhao W(79)	Adequate	Unclear	Inadequate	Death: Adequate<br>ARDS: Adequate	ARDS: Unclear	Inadequate*	Adequate	Unclear

*Patients followed-up for at least 2 weeks, until discharge or death

Table S7. Patient demographics and clinical characteristics at study entry in peer-reviewed studies

Study	Patients No.	Male No. (%)	Age years	Severity of COVID-19 No. (%)	Patients with comorbidities No. (%)	Respiratory system disease No. (%)	Cardiovascular disease No. (%)	Chronic kidney disease No. (%)	Diabetes No. (%)	Hypertension No. (%)	Cancer No. (%)	Co-infection No. (%)
Cao B(30)	199	120 (60.3)	58	199 (100) §	42 (21.1)	-	13 (6.5)	-	23 (11.6)	-	6 (3.0)	7 (3.5)
Cao J(31)	102	53(52.0)	54	102 (100) ‖	47 (46.1)	10 (9.8)	11 (10.8)	4 (3.9)	11 (10.8)	28 (27.5)	4 (3.9)	-
Chen J(32)	249	126 (50.6)	51	-	90 (36.1)	5 (2.0)	55 (21.7)	-	-	-	1 (0.4)	-
Chen N(4)	99	67 (67.7)	56	-	50 (50.5)	1 (1.0)	40 (40.4)	-	12 (12.1)	-	1 (1.0)	5 (5.1)
Chen T(33)	203	108 (53.2)	54	107 (52.7) ‡	88 (43.3)	8 (3.9)	25 (12.3)	8 (3.9)	16 (7.9)	43 (21.2)	7 (3.4)	-
Cui S(34)	81	37 (45.7)	60	81 (100) ‖	33 (40.7)	-	10 (12.3)	-	8 (9.9)	20 (24.7)	-	-
Fan Z(35)	148	75 (50.7)	50	-	-	-	-	-	-	-	-	-
Feng Y(36)	476	271 (56.9)	53	124 (26.1) ‡	205 (43.1)	22 (4.6)	5 (11.6)	4 (0.8)	49 (10.3)	113 (23.7)	12 (2.5)	-
Gao L(37)	54	24 (44.4)	60	54 (100) ‖	-	2 (3.7)	9 (16.7)		8 (14.8)	12 (22.2)	-	-
Grasselli G(38)	1591	1304 (82.0)	63	1591 (100) ‖	1043 (65.6)	42 (2.6)	223 (14.0)	36 (2.3)	180 (11.3)	509 (32.0)	81 (5.1)	-
Grein J(39)	53	40 (75.5)	64	53 (100) ‖	36 (67.9)	6 (11.3)	-	-	9 (17.0)	13 (24.5)	-	-
Guan Wei-jie(40)	1590	904/1578	49	254 (16.0) †	399 (25.1)	24 (1.5)	8 (5.6)	269 (16.9)	130 (8.2)	269 (16.9)	130 (8.2)	-
Guan Wei-jie(41)	1099	640 (58.2)	47	173 (15.7) †	261 (23.7)	12 (1.1)	42 (3.8)	8 (0.7)	81 (7.4)	165 (15.0)	10 (0.9)	-
Guo T(42)	187	91 (48.7)	58	-	-	4 (2.1)	29 (15.5)	6 (3.2)	28 (15.0)	61 (32.6)	13 (7.0)	-
Han H(43)	273	97 (35.5)	-	75 (27.5) §	-	-	-	-	-	-	-	-
Jin X(44)	651	331 (50.8)	-	64 (9.8) ‖	178 (27.3)	1 (0.2)	5 (0.8)	6 (0.9)	48 (7.4)	100 (15.4)	6 (0.9)	-
Klok FA(45)	184	139 (75.5)	64	-	-	-	-	-	-	-	5 (2.7)	-
Li R(46)	225	120 (53.3)	50	37 (16.4) ‡	-	-	-	-	-	47 (20.9)	-	-
Li X(47)	548	279 (50.9)	60	269 (49.1) †	-	23 (4.2)	34 (6.2)	10 (1.8)	83 (15.1)	166 (30.3)	24 (4.4)	42 (7.7)
Lian J(48)	788	381 (48.4)	-	78 (9.9) ‡	218 (27.7)	3 (0.4)	11 (1.4)	7 (0.9)	57 (7.2)	126 (16.0)	6 (0.8)	-
Liu Kay(49)	56	31 (55.5)	-	4 (6.0) ‖	-	-	2 (3.6)	1 (1.8)	4 (7.1)	10 (17.9)	-	9 (16.1)

Liu Kui(50)	137	61 (44.5)	57	137 (100) §	27 (19.7)	2 (1.5)	10 (7.3)	-	14 (10.2)	13 (9.5)	2 (1.5)	-
Liu W(51)	78	39 (50.0)	38	8 (10.3) ‡	29 (37.2)	2 (2.6)	-	-	5 (6.4)	8 (10.3)	4 (5.1)	0
Qian G(52)	91	37 (40.6)	50	9 (9.9) ‡	-	-	3 (3.3)	-	8 (8.8)	15 (16.5)	-	-
Shi H(53)	81	42 (51.9)	49	-	21 (25.9)	9 (11.1)	14 (17.3)	3 (3.7)	10 (12.3)	12 (14.8)	4 (4.9)	-
Shi S(54)	416	205 (49.3)	64	-	-	12 (2.9)	66 (15.9)	14 (3.4)	60 (14.4)	127 (30.5)	9 (2.2)	-
Tang Ning(55)	449	268 (59.7)	65	449 (100) ‡	272 (60.6)	-	-	-	-	-	-	-
Tang Ning(56)	183	98 (53.6)	54	-	75 (41.0)	4 (2.2)	-	-	-	-	-	-
Tian S(57)	262	127 (58.5)	47	46 (17.6) §	-	-	-	-	-	-	-	-
Wang D(58)	138	75 (54.3)	56	-	64 (56.4)	4 (2.9)	27 (19.6)	4 (2.9)	14 (10.1)	43 (31.2)	10 (7.2)	-
Wang Lang(59)	339	166 (49.0)	69	239 (70.5) §	-	21 (6.2)	74 (21.8)	12 (3.5)	54 (15.9)	138 (40.7)	15 (4.4)	143 (42.2)
Wang Luwen(60)	116	67 (57.8)	54	57 (49.1) §	-	-	7 (6.0)	5 (4.3)	18 (15.5)	43 (37.1)	12 (10.3)	-
Wang Yang(61)	344	179 (52.0)	64	344 (100) ‡	-	16 (4.7)	40 (11.6)	-	64 (18.6)	141 (41.0)	-	-
Wang Yuhui(62)	90	33 (36.7)	45	-	-	-	-	-	-	-	-	-
Wang Z(63)	69	32 (46.0)	42	-	-	6 (8.7)	8 (12.0)	-	7 (10.0)	9 (13.0)	4 (16)	-
Wu C(64)	201	128 (63.7)	51	-	-	5 (2.5)	8 (4.0)	2 (1.0)	22 (10.9)	39 (19.4)	1 (0.5)	1 (0.5)
Wu J(65)	80	39 (48.8)	46	3 (3.8) ‡	-	1 (1.3)	25 (31.3)	1 (1.3)	5 (6.3)	-	1 (1.3)	12 (15.0)
Xu XW(66)	62	35 (56.5)	41	-	20 (32.3)	1 (1.6)	1 (1.6)	1 (1.6)	1 (1.6)	5 (8.1)	-	-
Yang W(67)	149	81 (54.4)	45	0§	-	1 (0.7)	28 (18.8)	-	9 (6.0)	-	2 (1.3)	-
Yang Xiaobo(68)	52	35 (67.3)	59	52 (100) §	21 (40.4)	4 (7.7)	12 (23.1)	-	9 (17.3)	-	2 (3.8)	8 (15.4)
Yang Xiaobo(69)	1476	776 (52.6)	-	-	-	-	-	-	-	-	-	-
Zhang J(70)	663	321 (48.4)	55.6	409 (61.7) §	247 (37.3)	51 (7.7)	-	-	-	-	14 (2.1)	-
Zhang L(71)	343	169 (49.3)	62	-	120 (35.0)	8 (2.3)	27 (7.9)	4 (1.2)	47 (13.7)	76 (22.2)	9 (2.6)	-
Zhou F(72)	191	119 (62.3)	56	119 (62.3) ‡	91 (47.6)	6 (3.1)	15 (7.9)	2 (1.0)	36 (18.8)	58 (30.4)	2 (1.0)	28 (14.7)

† = American Thoracic Society definition; ‡ = National Health Commission of the People's Republic of China definition; § = World Health Organization definition, ‖ = other definition

Table S8. Treatment provided in peer-reviewed studies

Study	Patients No.	Antiviral therapy No. (%)	Antibiotic therapy No. (%)	Glucocorticoids No. (%)	Immunoglobulin therapy No. (%)	Supplemental oxygen No. (%)	NIV No. (%)	IMV No. (%)	ECMO No. (%)	CRRT No. (%)
Cao B(1)	199	95 (47.7)	189 (95.0)	67 (33.7)	-	170 (85.4)	29 (14.6)	32 (16.1)	4 (2.0)	9 (4.5)
Cao J(2)	102	102 (100)	101 (99.0)	51 (50.0)	11 (10.8)	76 (74.5)	4 (4.9)	14 (13.7)	3 (2.9)	6 (5.9)
Chen J(3)	249	-	-	32 (12.9)	-	-	-	-	-	-
Chen N(4)	99	75 (75.8)	70 (70.7)	19 (19.2)	27 (27.3)	75 (75.8)	13 (13.1)	4 (84.0)	3 (3.0)	9 (9.1)
Chen T(5)	203	131 (64.5)	-	107 (52.7)	11 (5.4)	123 (60.6)	-	-	-	-
Cui S(6)	81	81 (100)	-	-	-	-	-	-	-	-
Fan Z(7)	148	-	-	-	-	-	-	-	-	-
Feng Y(8)	476	286 (60.1)	319 (67.0)	127 (26.7)	-	-	34 (7.1)	39 (8.2)	4 (0.8)	-
Gao L(9)	54	-	-	-	-	-	-	-	-	-
Grasselli G(10)	1591	-	-	-	-	13 (1.0)	137 (11.0)	1150 (88.0)	-	-
Grein J(11)	53	53 (100)	-	-	-	15 (28.3)	2 (3.7)	30 (88.0)	4 (12.0)	-
Guan Wei-jie(12)	1590	-	-	-	-	-	-	50 (3.1)	-	-
Guan Wei-jie(13)	1099	393 (35.8)	637 (58.0)	204 (18.6)	144 (13.1)	454 (41.3)	25 (2.3)	56 (5.1)	5 (0.5)	9 (0.8)
Guo T(14)	187	166 (88.8)	183 (97.9)	106 (56.7)	21 (11.2)	-	-	-	-	-
Han H(15)	273	-	-	-	-	-	-	-	-	-
Jin X(16)	651	546 (83.9)	277 (42.5)	74 (11.4)	-	-	-	17 (2.6)	0	0
Klok FA(17)	184	-	-	-	-	-	-	-	-	23 (12.5)
Li R(18)	225	-	-	-	-	-	-	-	-	-
Li X(19)	548	-	-	341 (62.2)	213 (38.9)	228 (41.6)	78 (14.2)	25 (4.6)	-	2 (0.4)
Lian J(20)	788	668 (84.8)	-	100 (12.7)	62 (7.9)	-	7 (0.9)	11 (1.4)	0	0
Liu Kay(21)	56	53 (94.6)	40 (71.4)	-	9 (16.1)	54 (96.4)	3 (5.4)	7 (12.5)	2 (3.6)	5 (8.9)
Liu Kui(22)	137	105 (76.6)	119 (86.9)	40 (29.2)	44 (32.1)	119 (86.9)	34 (24.8)	-	-	-

Liu W(23)	78	78 (100)	78 (100)	45 (57.7)	14 (17.9)	78 (100)	5 (6.4)	0	0	0
Qian G(24)	91	-	-	-	-	-	-	-	-	-
Shi H(25)	81	-	-	-	-	-	-	-	-	-
Shi S(26)	416	403 (96.9)	235 (56.5)	304 (73.1)	259 (62.3)	367 (88.2)	51 (12.3)	32 (7.7)	-	2 (0.5)
Tang Ning(27)	449	-	-	-	-	-	-	-	-	-
Tang Ning(28)	183	-	-	-	-	-	-	-	-	-
Tian S(29)	262	-	-	-	-	-	-	-	-	-
Wang D(30)	138	124 (89.9)	-	62 (44.9)	-	106 (76.8)	15 (10.9)	17 (12.3)	4 (82.9)	2 (1.4)
Wang Lang(31)	339	-	-	-	-	-	-	-	-	-
Wang Luwen(32)	116	-	-	-	-	-	-	-	-	-
Wang Yang(33)	344	-	-	-	-	-	34 (9.9)	100 (29.1)	-	-
Wang Yuhui(34)	90	-	-	-	-	-	-	-	-	-
Wang Z(35)	69	66 (98.5)*	66 (98.5)*	10 (14.9)*	-	-	-	-	-	-
Wu C(36)	201	170 (84.6)	196 (97.5)	62 (30.8)	-	159 (79.1)	61 (30.3)	6 (3.0)	1(0.5)	-
Wu J(37)	80	80 (100)	73 (91.3)	-	16 (20.0)	-	35 (43.8)	0	0	-
Xu XW(38)	62	55 (88.7)	28 (45.2)	16 (25.8)	16 (25.8)	-	-	1 (1.8)	-	-
Yang W(39)	149	140 (94.0)	34 (22.8)	5 (3.4)	19 (12.8)	134 (89.9)	2 (1.3)	0	0	-
Yang Xiaobo(40)	52	23 (44.2)	49 (94.2)	30 (57.7)	28 (53.8)	52 (100)	29 (55.8)	22 (42.3)	6 (11.5)	9 (17.3)
Yang Xiaobo(41)	1476	-	-	-	-	-	-	-	-	-
Zhang J(42)	663	-	-	-	-	-	-	-	-	-
Zhang L(43)	343	-	-	-	-	-	-	-	-	-
Zhou F(44)	191	41 (21.5)	181 (94.8)	57 (29.8)	46 (24.1)	-	26 (13.6)	32 (16.8)	3 (1.6)	10 (5.2)

*Data available only for 67 patients. CRRT: chronic renal replacement therapy; ECMO: extra-corporeal membrane oxygenation; IMV: invasive mechanical ventilation, NIV: non-invasive ventilation

Table S9. Patient demographics and clinical characteristics at study entry in not peer-reviewed studies

**Study**	**Patients No.**	**Male No. (%)**	**Age years**	**Severity of COVID-19 No. (%)**	**Patients with Comorbidities No. (%)**	**Respiratory system disease No. (%)**	**Cardiovascular disease No. (%)**	**Chronic kidney disease No. (%)**	**Diabetes No. (%)**	**Hypertension No. (%)**	**Cancer No. (%)**
Benelli G(45)	411	359 (66.6)	71	-	256 (62.3)	48 (11.7)	93 (22.6)	22 (5.6)	76 (18.5)	193 (47.0)	33 (8.0)
Chen C(46)	236	110 (46.6)	-	27 (11.4)§	-	-	-	-	27 (11.4)	66 (28.0)	-
Chen Xiaoping(47)	123	50 (40.7)	51	33 (26.8)§	35 (28.5)	-	8 (6.5)	-	12 (9.8)	19 (15.4)	5 (4.1)
Chen Xu(48)	291	145 (49.8)	46	50 (17.2)§	93 (32.0)	10 (3.4)	20 (6.9)	2 (0.7)	22 (7.6)	8 (2.7)	2 (0.7)
Cheng Y(49)	710	374 (52.7)	63	252 (35.5)‡	-	-	-	-	-	-	-
Cummings MJ(50)	257	170 (66.1)	62	257 (100)‖	212 (82.5)	45 (17.5)	-	37 (14.4)	92 (35.8)	162 (63.0)	18 (7.0)
Fan L(51)	55	30	47	8 (14.5)‡	19 (34.5)	-	3 (5.5)	-	8 (14.4)	8 (14.5)	-
Feng Z(52)	564	284	47	69 (12.2)‖	132 (21.8)	16 (2.8)	-	3 (0.5)	45 (8.0)	82 (14.5)	4 (0.7)
Fu L(53)	200	99 (49.5)	-	109 (54.5)‖	161 (80.5)	8 (4.0)	16 (8.0)	-	137 (68.5)	101 (50.5)	-
Giorgi Rossi P(54)	2653	1328	-	-	-	128 (4.8)	168 (6.3)	59 (2.2)	284 (10.7)	430 (16.2)	301 (11.3)
Hu L(55)	323	166 (51.4)	61	172 (53.3)§	-	29 (9.0)	41 (12.7)	7 (2.2)	47 (14.6)	105 (32.5)	5 (1.5)
Jiang X(56)	55	27	45	8 (14.5)‡	29 (52.7)	-	3 (5.5)	1 (1.8)	9 (16.4)	17 (31.0)	2 (3.6)
Joharatnam-Hogan N(57)	52	31	-	-	52 (100)	-	-	-	-	-	26 (50.0)
Liu J(58)	64	23 (35.9)	35	1 (1.6) ‖	8 (12.5)	-	-	-	1 (1.6)	3 (4.7)	-
Liu Qibin(59)	504	259	-	-	262 (52.0)	-	-	-	-	-	-
Liu Qibin(60)	340	172	-	-	130 (38.2)	11 (3.2)	24 (7.1)	-	8 (2.4)	84 (24.7)	-
Liu Yanli(61)	109	59 (54.1)	55	-	-	4 (3.7)	13 (11.9)	10 (9.2)	12 (11.0)	37 (33.9)	-
Liu Youbin(62)	291	133 (45.7)	48	-	-	-	12 (4.1)	-	22 (7.6)	54 (18.6)	-
Lu X(63)	244	128	62	244 (100)§	-	12 (4.9)	28 (11.5)	-	44 (18.0)	95 (38.9)	-
Magagnoli J(64)	368	368	-	-	-	94 (25.5)	-	92 (25.0)	249 (67.7)	-	66 (17.9)
Qi D(65)	267	149 (55.8)	48	50 (18.7)†	41 (15.4)	25 (9.4)	13 (4.9)	-	26 (9.7)	20 (7.5)	-
Qiu C(66)	104	49 (47.1)	43	16 (15.4)‖	-	1 (1.0)	7 (6.7)	-	12 (11.5)	15 (14.4)	-
Rubin SJ(67)	54	29	54	-	-	7 (13.0)	3 (5.6)	3 (5.6)	7 (13.0)	14 (25.9)	-
Wang Z(68)	538	285		88 (16.4)‡	-	-	-	-	-	-	

Wen Y(69)	417	197 (47.2)	45	36 (8.6)‖	-	-	-	-	-	-	-
Wu C(70)	188	119 (63.3)	52	-	64 (34.0)	2 (1.1)	12 (6.4)	3 (1.6)	20 (10.6)	38 (20.2)	-
Wu Y(71)	297	147 (49.5)	-	83 (27.9)§	-	-	-	-	-	-	-
Xiao G(72)	287	160	62	124 (43.2)†	-	16 (5.6)	33 (11.5)	-	45 (15.7)	-	8 (2.8)
Xu H(73)	53	28 (52.8)	-	8 (15.1)‡	-	3 (5.7)	6 (11.3)	-	8 (15.1)	8 (15.1)	-
Xu S(74)	355	193 (54.4)	-	131 (36.9)§	-	-	-	-	147 (41.4)	125 (35.2)	-
Xu Y(75)	69	35 (50.7)	57	25 (36.2)§	0	0	0	0	0	0	0
Yan S(76)	168	81 (48.2)	51	36 (21.4)‡	-	12 (7.1)	12 (7.1)	1 (0.6)	12 (7.1)	24 (14.3)	2 (1.2)
Zeng L(77)	338	-	-	-	-	-	-	-	-	-	
Zhang G(78)	221	108 (48.9)	55	55 (24.9)§	78 (35.3)	6 (2.7)	37 (16.7)	6 (2.7)	22 (10.0)	54 (24.4)	9 (4.1)
Zhao W(79)	77	34 (44.2)	52	20 (26.0)§	24 (31.2)	6 (7.8)	11 (14.3)	5 (6.5)	6 (7.8)	16 (20.8)	4 (5.2)

*Data available for 1578 patients ; † = American Thoracic Society definition; ‡ = National Health Commission of the People's Republic of China definition; § = World Health Organization definition, ‖ = other definition

Table S10. Treatment provided in not peer-reviewed studies

Study	Patients No.	Antiviral therapy No. (%)	Antibiotic therapy No. (%)	Glucocorticoids No. (%)	Immunoglobulin therapy No. (%)	Supplemental oxygen No. (%)	NIV No. (%)	IMV No. (%)	ECMO No. (%)	CCRT No. (%)
Benelli G(45)	411	174 (42.3)	-	-	-	-	112 (27.3)	-	-	-
Chen C(46)	236	236 (100)	-	-	-	-	-	-	-	-
Chen Xiaoping(47)	123	74 (60.2)	123 (100)	61 (49.6)	-	-	-	-	-	-
Chen Xu(48)	291	285 (97.9)	-	67 (23.0)	-	-	-	-	-	-
Cheng Y(49)	710	-	-	-	-	-	-	-	-	-
Cummings MJ(50)	257	23 (8.9)	229 (89.1)	68 (26.6)	-	127 (49.4)	3 (1.2)	201 (78.2)	5 (1.9)	75 (29.2)
Fan L(51)	55	53 (96.4)	29 (52.7)	7 (12.7)	-	-	-	-	-	-
Feng Z(52)	564	503 (89.2)	-	-	-	-	-	-	-	-
Fu L(53)	200	-	-	-	-	-	-	-	-	-
Giorgi Rossi P(54)	2653	-	-	-	-	-	-	-	-	-
Hu L(55)	323	-	304 (94.1)	196 (60.7)	-	-	105 (32.5)	34 (10.5)	-	72 (22.3)
Jiang X(56)	55	55 (100)	29 (52.7)	6 (10.9)	-	29 (52.7)	-	7 (12.7)	1 (1.8)	-
Joharatnam-Hogan N(57)	52	-	-	-	-	-	-	-	-	-
Liu J(58)	64	64 (100)	55 (85.9)	7 (10.9)	23 (35.9)	32 (50.0)	2 (3.1)	-	-	-
Liu Qibin(59)	504	359 (71.2)	-	230 (45.6)	152 (30.2)	-	-	-	-	-
Liu Qibin(60)	340	-	-	-	-	-	-	-	-	-
Liu Yanli(61)	109	107 (98.2)	106 (97.2)	43 (39.4)	61 (56.0)	28 (25.7)	-	-	-	-
Liu Youbin(62)	291	-	-	-	-	-	-	31 (10.7)	4 (1.4)	5 (1.7)
Lu X(63)	244	244 (100)	184 (75.4)	151 (61.9)	92 (37.7)	22 (9.0)	82 (33.6)	-	-	-
Magagnoli J(64)	368	-	-	-	-	-	-	44 (12.0)	-	-
Qi D(65)	267	267 (100)	43 (16.1)	38 (14.2)	35 (13.1)	91 (34.1)	35 (13.1)	5 (1.9)	0	0
Qiu C(66)	104	103 (99.0)	21 (20.2)	9 (14.0)	14 (13.5)	-	-	-	-	
Rubin SJ(67)	54	-	-	-	-	-	-	-	-	-
Wang Z(68)	538	-	-	-	-	-	-	-	-	-

Wen Y(69)	417	-	-	-	-	-	-	-	-	
Wu C(70)	188	158 (84.0)	185 (98.4)	59 (31.4)	-	-	-	-	-	
Wu Y(71)	297	-	-	-	-	-	-	-	-	
Xiao G(72)	287	-	-	-	-	-	-	-	-	-
Xu H(73)	53	53 (100)	14 (26.4)	-	-	53 (100)	8 (15.1)	3 (5.7)	-	4 (7.5)
Xu S(74)	355	-	-	-	-	-	-	-	-	-
Xu Y(75)	69	38 (55.1)	31 (44.9)	6 (8.7)	-	35 (50.7)	3 (4.3)	2 (2.9)	0	-
Yan S(76)	168	155 (92.3)	119 (70.8)	27 (16.1)	-	97 (57.7)	13 (7.7)	12 (7.1)	-	-
Zeng L(77)	338	-	-	-	-	-	-	-	-	5 (1.5)
Zhang G(78)	221	196 (88.7)	-	64 (29.0)	-	-	27 (12.2)	26 (11.8)	10 (4.5)	-
Zhao W(79)	77	-	-	-	-	-	-	-	-	-

CRRT: chronic renal replacement therapy; ECMO: extra-corporeal membrane oxygenation; IMV: invasive mechanical ventilation, NIV: non-invasive ventilation

Table S11. Clinical outcomes of patients in peer-reviewed studies

Study	Patients No.	Clinical improvement, recovery or discharge No. (%)	Still in hospital at end of study No. (%)	Admission to ICU No. (%)	All-cause mortality No. (%)	ARDS No. (%)	Acute cardiac injury No. (%)	Venous thromboembolism No. (%)	Shock No. (%)	AKI No. (%)	Coagulopathy No. (%)
Cao B(30)	199	148 (74.4)	-	-	44 (22.1)	39 (19.6)	-	-	4 (2.0)	9 (4.5)	2 (1.0)
Cao J(31)	102	85 (83.3)	0	18 (17.6)	17 (16.7)	1 (1.0)	4 (3.9)	-	-	-	-
Chen J(32)	249	215 (86.3)	32 (12.9)	22 (8.8)	2 (0.8)	8 (3.2)	-	-	-	-	-
Chen N(4)	99	31 (31.3)	57 (57.6)	23 (23.2)	11(11.1)	17 (17.2)	-	-	4 (4.0)	3 (3.0)	-
Chen T(33)	203	177 (87.2)	0	-	26 (12.8)	18 (8.9)	4 (2.0)	-	2 (1.0)	-	1 (0.5)
Cui S(34)	81	64 (79.0)	9 (11.1)	81 (100.0)	8 (9.9)	-	-	20 (24.7)	-	-	-
Fan Z(35)	148	92 (62.2)	55 (37.2)	-	1 (0.7)	-	-	-	-	-	-
Feng Y(36)	476	403 (84.7)	23 (4.8)	-	38 (8.0)	-	-	-	-	-	-
Gao L(37)	54	-	-	-	18 (33.3)	-	-	-	-	-	-
Grasselli G(38)	1591	256 (16.2)#	920/581	1591 (100.0)	405/1581	-	-	-	-	-	-
Grein J(39)	53	25 (47.2)	-	-	7 (13.2)	2 (3.8)	-	3 (5.7)	2 (3.8)	3 (5.7)	-
Guan Wei-jie(40)	1590	-	-	99 (6.2)	50 (3.1)		-	-	-	-	-
Guan Wei-jie(41)	1099	55 (5.0)	-	55 (5.0)	15 (1.4)	37 (3.4)	-	-	12 (1.1)	6 (0.5)	1 (0.1)
Guo T(42)	187	144 (77.0)	0	-	43 (23.0)	46 (24.6)	-	-	-	18 (9.6)	42 (22.5)
Han H(43)	273	246 (90.1)	3 (1.1)	-	24 (8.8)	-	-	-	-	-	-
Jin X(44)	651	-	-	17 (2.6)	-	17 (2.6)	-	-	2 (0.3)	-	-
Klok FA(45)	184	22 (12.0)	139 (75.5)	184 (100.0)	23 (12.5)	-	-	28 (15.2)	-	-	-
Li R(46)	225	20 (8.9)	-	-	2 (0.9)	-	-	-	-	-	-
Li X(47)	548	287 (52.4)	168 (30.7)	-	90 (16.4)	210 (38.3)	119 (21.7)	-	-	95 (17.3)	41 (7.5)
Lian J(48)	788	322 (40.9)	446 (56.6)	22 (2.8)	0	58 (7.4)	-	-	2 (0.3)	13 (1.6)	-

Liu Kay(49)	56	53 (94.6)	-	-	3 (5.4)	6 (10.7)	7 (12.5)	-	3 (5.4)	-	-
Liu Kui(50)	137	44 (32.1)	77 (56.2)	-	16 (11.7)	-	-	-	-	-	-
Liu W(51)	78	67 (85.9)	-	-	2 (2.6)	-	-	-	-	-	-
Qian G(52)	91	31 (34.1)	60 (65.9)	-	0	-	-	-	-	-	-
Shi H(53)	81	62 (76.5)	16 (19.8)	-	3 (3.7)	-	-	-	-	-	-
Shi S(54)	416	40 (9.6)	319 (76.9)	-	57 (13.7)	97 (23.3)	82 (19.7)	-	-	8 (1.9)	12 (2.9)
Tang Ning(55)	449	315 (70.2)	-		134 (29.8)	-	-	-		-	-
Tang Ning(56)	183	78 (42.6)	84 (45.9)	-	21 (11.5)	-	-	-	-	-	16 (8.7)
Tian S(57)	262	45 (17.2)	214 (81.7)	-	3 (1.1)	-	-	-	-	-	-
Wang D(58)	138	47 (34.1)	85 (61.6)	36 (26.1)	6 (4.3)	27 (19.6)	10 (7.2)	-	12 (8.7)	5 (3.6)	-
Wang Lang(59)	339	91 (26.8)	183 (54.0)	-	65 (19.2)	71 (20.9)	70 (20.6)	-	8 (2.4)	27 (8.0)	-
Wang Luwen(60)	116	-	-	11 (9.5)	7 (6.0)	11 (9.5)	-	-	-	-	-
Wang Yang(61)	344	211 (61.3)	0	344 (100.0)	133 (38.7)	145 (42.2)	111 (32.3)	-	114 (33.1)	86 (25.0)	71 (20.6)
Wang Yuhui(62)	90	70 (77.8)	17 (18.9)	-	2 (2.2)	-	-	-	-	-	-
Wang Z(63)	69	18 (26.8) *	44 (65.7) *	-	5 (0.4) *	-	-	-	-	-	-
Wu C(64)	201	144 (71.6)	131 (14.9)	53 (26.4)	44 (21.9)	84 (41.8)	-	-	-	-	-
Wu J(65)	80	21 (26.3)	61 (76.3)	-	0	10 (12.5)	-	-	-	2 (2.5)	-
Xu XW(66)	62	1 (1.6)	61 (98.4)	1 (1.6)	0	1 (1.6)	-	-	-	-	-
Yang W(67)	149	73 (49.0)	76 (51.0)	0	0	0	-	-	0	0	-
Yang Xiaobo(68)	52	8 (15.4)	12 (23.1)	52 (100.0)	32 (61.5)	35 (67.3)	12 (23.1)	-	-	15 (28.8)	-
Yang Xiaobo(69)	1476	1238 (83.9)	0	-	238 (16.1)	-	-	-	-	-	-
Zhang J(70)	663	251 (37.9)	-	-	25 (3.8)	-	-	-	-	-	-
Zhang L(71)	343	-		-	13 (3.8)	-	-	-	-	-	-
Zhou F(72)	191	137 (71.7)	0	50 (26.2)	54 (28.3)	59 (30.9)	33 (17.3)	-	38 (19.9)	28 (14.7)	37 (19.4)

*Data available for 67 patients. #Data available for 1581 patients. AKI: acute kidney injury; ARDS: acute respiratory distress syndrome; ICU: intensive care unit.

Table S12. Clinical outcomes of patients in not peer-reviewed studies

Study	Patients No.	Clinical improvement, recovery or discharge No. (%)	Still in hospital at end of study No. (%)	Admission to ICU No. (%)	All-cause mortality No. (%)	ARDS No. (%)	Shock No. (%)	Acute kidney injury No. (%)	Acute cardiac injury No. (%)	Coagulopathy No. (%)
Benelli G(45)	411	-	-	28 (6.8)	72 (17.5)	-	-	-	-	-
Chen C(46)	236	-	-	-	0	-	-	-	-	-
Chen Xiaoping(47)	123	110 (89.4%)	8 (6.5)	-	5 (4.1)	-	-	-	-	-
Chen Xu(48)	291	159 (54.6)	130 (44.7)	-	2 (0.7)	-	-	-	-	-
Cheng Y(49)	710	-	-	-	89 (12.5)	-	-	22 (3.1)	-	-
Cummings MJ(50)	257	45 (17.5)	122 (47.5)	-	86 (33.4)	-	-	-	-	-
Fan L(51)	55	55 (100)	0	-	0	2 (3.6)	-	-	-	-
Feng Z(52)	564	-	-	-	2 (0.3)	-	-	-	-	-
Fu L(53)	200	-	-	-	34 (17.0)	-	-	-	-	-
Giorgi Rossi P(54)	2653	-	-	-	217 (8.2)	-	-	-	-	-
Hu L(55)	323	252 (78.0)	36 (11.1)	-	35 (10.8)	13 (4.0)	43 (13.3)	17 (5.3)	24 (7.4)	-
Jiang X(56)	55	55 (100)	0	-	0	4 (7.3)	1 (1.8)	3 (5.5)	1 (1.8)	-
Joharatnam-Hogan N(57)	52	-	-	-	12 (23.1)	-	-	-	-	-
Liu J(58)	64	34 (53.1)	30 (46.9)	0	0	-	-	-	-	-
Liu Qibin(59)	504	-	-	-	79	-	-	-	-	-
Liu Qibin(60)	340	310 (91.2)	0	-	30 (8.8)	-	-	-	-	-
Liu Yanli(61)	109	78 (71.6)	-	-	31 (28.4)	53 (48.6)	-	-	-	-
Liu Youbin(62)	291	-	-	26 (8.9)	1 (0.3)	3 (1.0)	-	-	15 (5.2)	-
Lu X(63)	244	-	-	-	-	87 (35.7)	71 (29.1)	51 (20.9)	67 (27.5)	41 (16.8)
Magagnoli J(64)	368	298 (81.0)	0	-	70 (19.0)	-	-	-	-	-
Qi D(65)	267	103 (38.6)	160 (22.5)	53 (19.9)	4 (1.5)	33 (12.4)	13 (4.9)	-	3 (1.1)	1 (0.4)
Qiu C(66)	104	40 (38.5)	63 (60.6)	9 (8.7)	1 (1.0)	12 (11.5)	2 (1.9)	2 (1.9)	3 (2.9)	-
Rubin SJ(67)	54	-	-	6 (11.1)	-	4 (7.4)	-	-	-	-

Wang Z(68)	538	364 (67.7)	171 (31.8)	-	3 (0.6)	-	-	-	-	-
Wen Y(69)	417	299 (71.7)	115 (27.6)	-	3 (0.7)	-	-	-	-	-
Wu C(70)	188	145 (77.1)	0	50 (26.6)	43 (22.9)	-	-	-	-	-
Wu Y(71)	297	-	-	-	17 (5.7)	-	-	-	-	-
Xiao G(72)	287	188 (65.5)	80 (27.9)	-	19 (6.6)	55 (19.2)	-	-	-	-
Xu H(73)	53	42 (79.2)	8 (15.1)	8 (15.1)	3 (5.7)	10 (18.9)	-	5 (9.4)	-	-
Xu S(74)	355	-	-	-	32 (9.0)	-	-	56 (15.8)	-	-
Xu Y(75)	69	4 (5.8)	-	-	1 (1.4)	5 (7.2)	3 (4.3)	-	5 (7.2)	-
Yan S(76)	168	160 (95.2)	2 (1.2)	-	6 (3.6)	17 (10.1)	7 (4.2)	6 (3.6)	4 (2.4)	2 (1.2)
Zeng L(77)	338	290 (85.8)	45 (13.3)	-	3 (0.9)	-	-	-	-	-
Zhang G(78)	221	42 (19.0)	168 (76.0)	44 (19.9)	12 (5.4)	48 (21.7)	15 (6.8)	10 (4.5)	17 (7.7)	-
Zhao W(79)	77	64 (83.1)	8 (10.4)	-	5 (6.5)	3 (3.9)	1 (1.3)	2 (2.6)	2 (2.6)	-

ARDS: acute respiratory distress syndrome; ICU: intensive care unit

Figure S1. PRISMA flow diagram

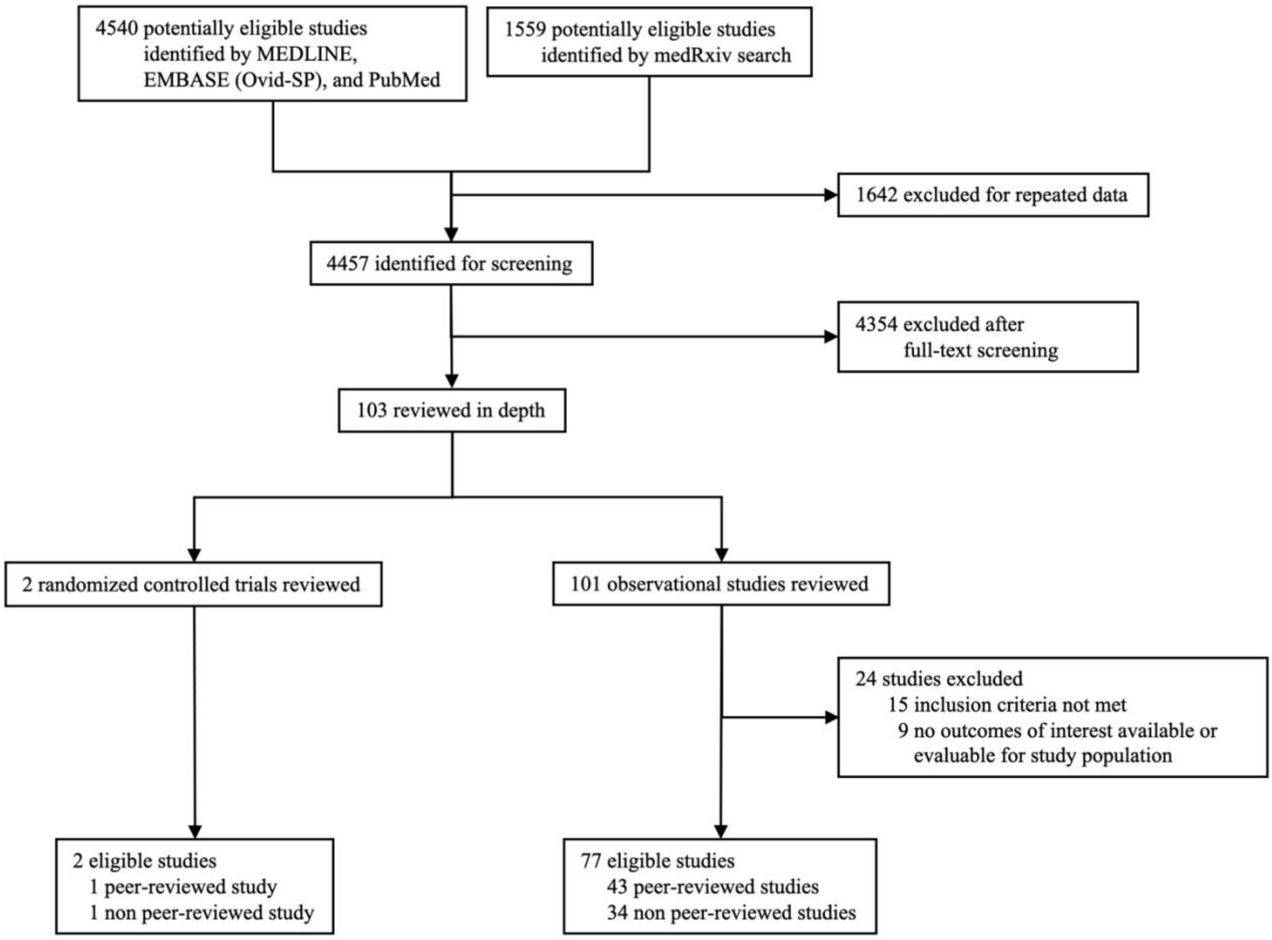

Figure S2. MINORS quality assessment for peer reviewed (panel A) and not peer-reviewed (panel B) studies

A

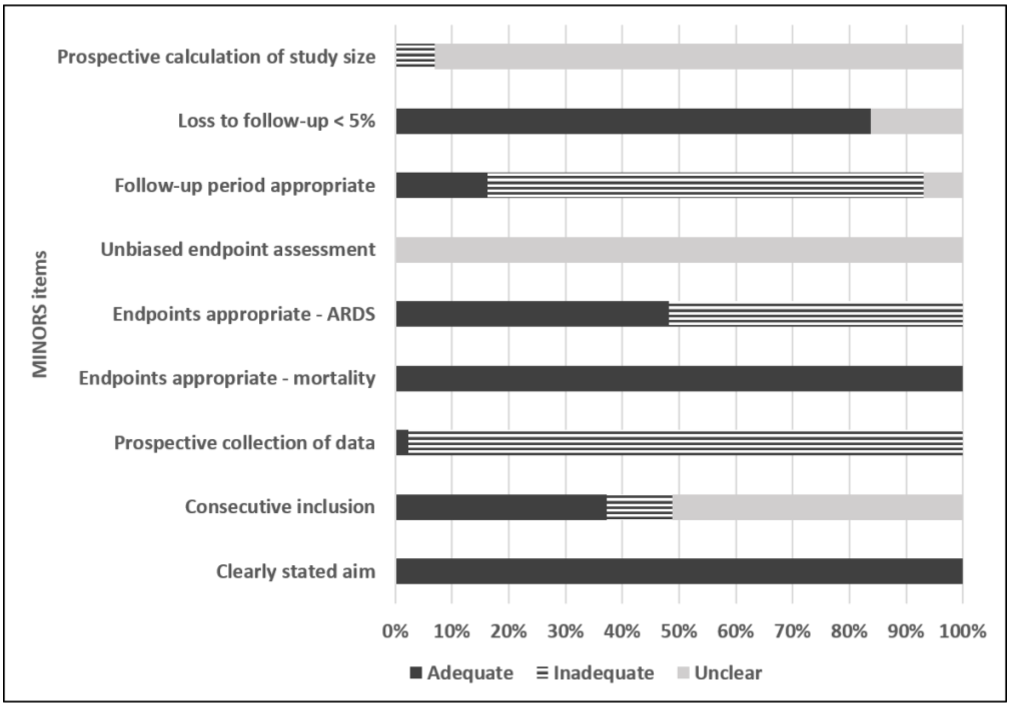

B

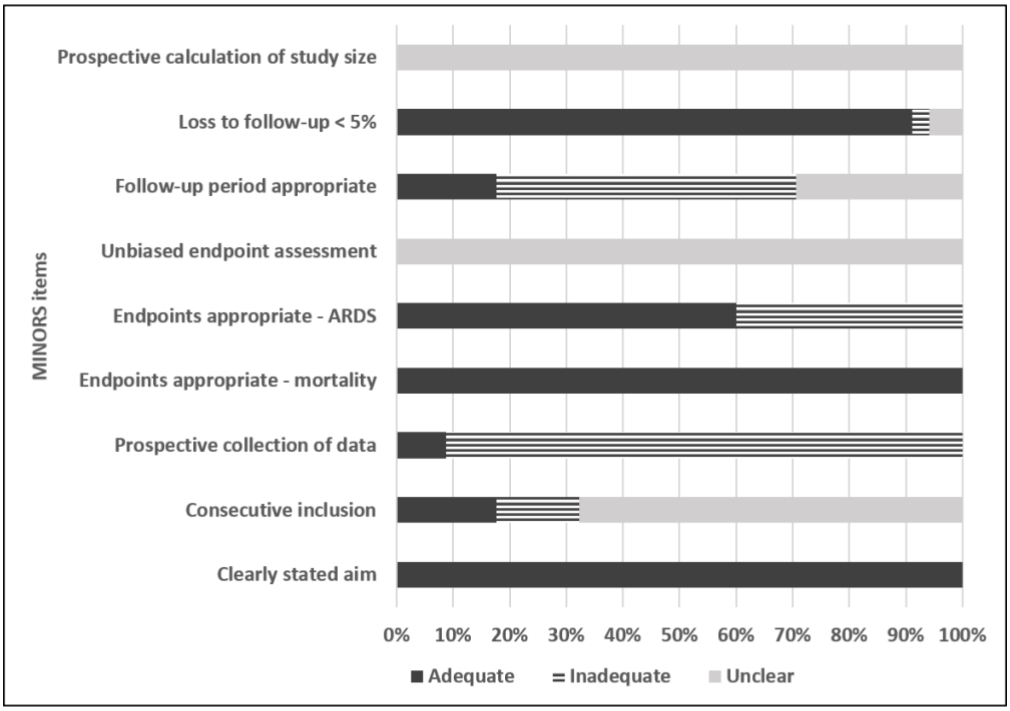

The item on unbiased endpoint assessment was deemed adequate for ARDS if an external independent adjudication committee was involved

ARDS, acute respiratory distress syndrome; MINORS, methodological index for non-randomized studies

Figure S3. All-cause mortality by subgroup of patients with COVID-19, peer-reviewed studies

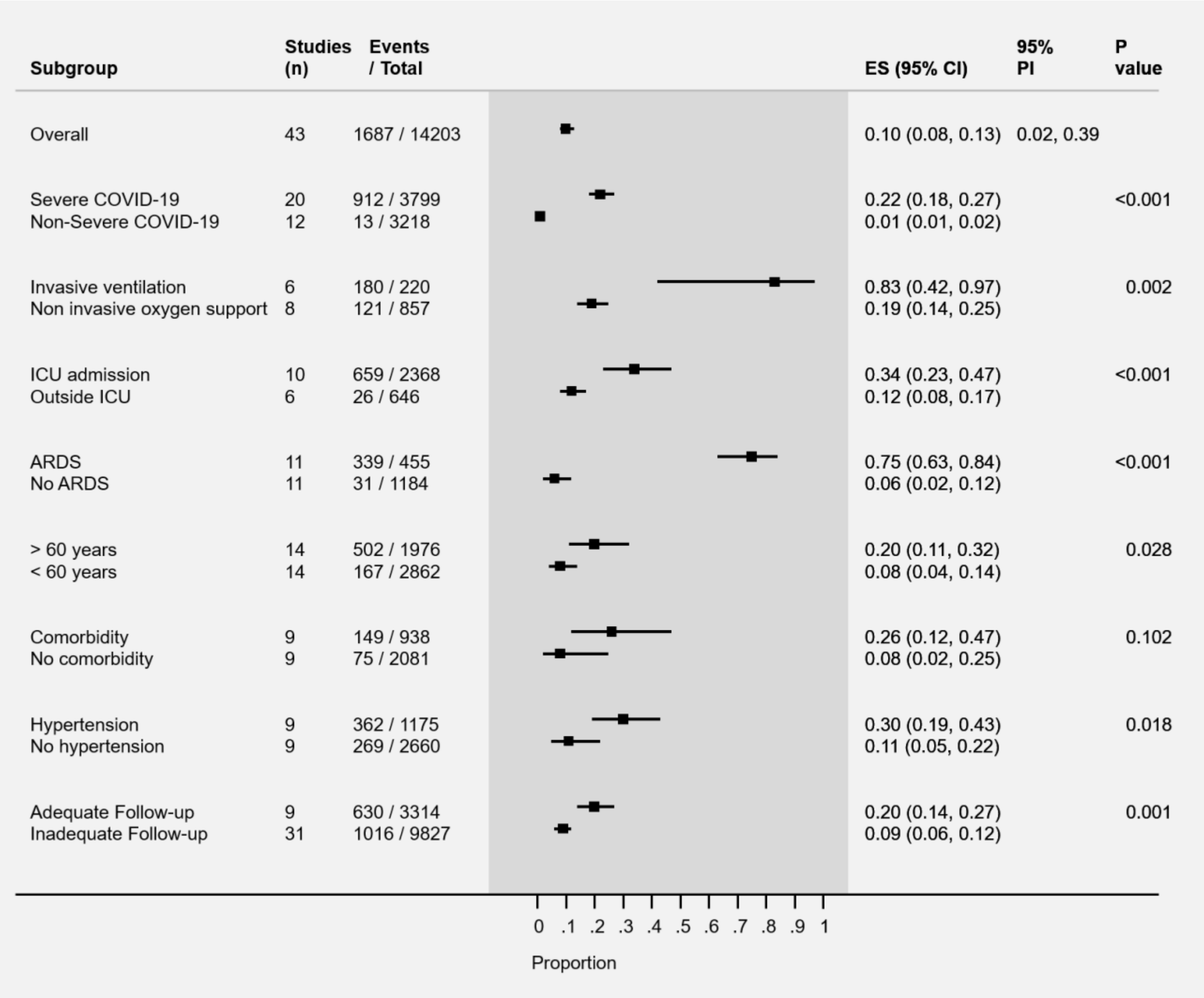

ARDS, acute respiratory distress syndrome; CI, confidence intervals; COVID-19, *Coronavirus* disease 2019; ES, estimates; ICU intensive care unit; PI, prediction intervals

Figure S4. ARDS by follow-up duration, peer-reviewed studies

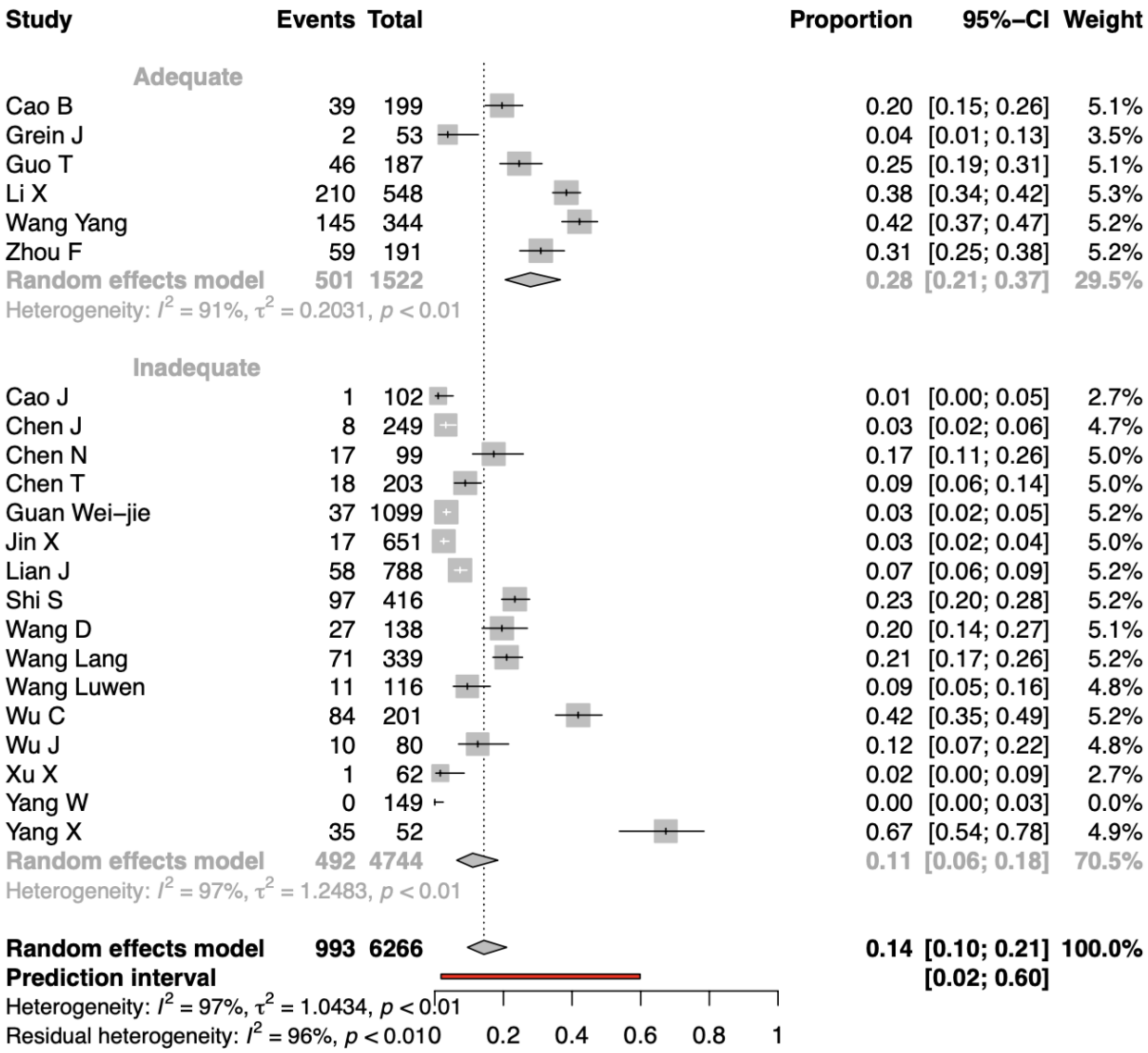

CI, confidence intervals

Figure S5. All-cause mortality in patients with COVID-19, not peer-reviewed studies

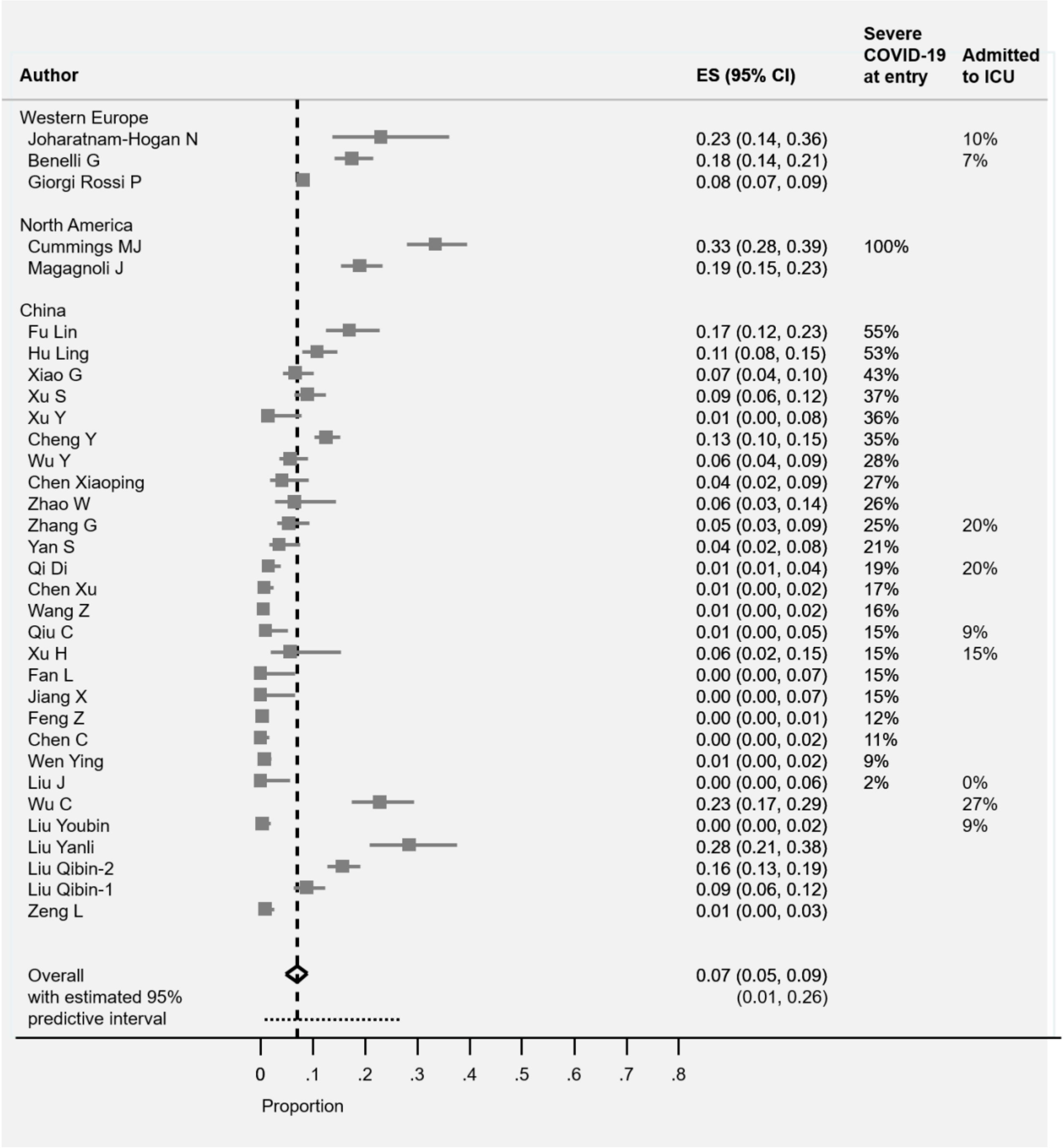

CI, confidence intervals; COVID-19, *Coronavirus* disease 2019; ES, estimates; ICU intensive care unit

Figure S6. ARDS in patients with COVID-19, not peer-reviewed studies

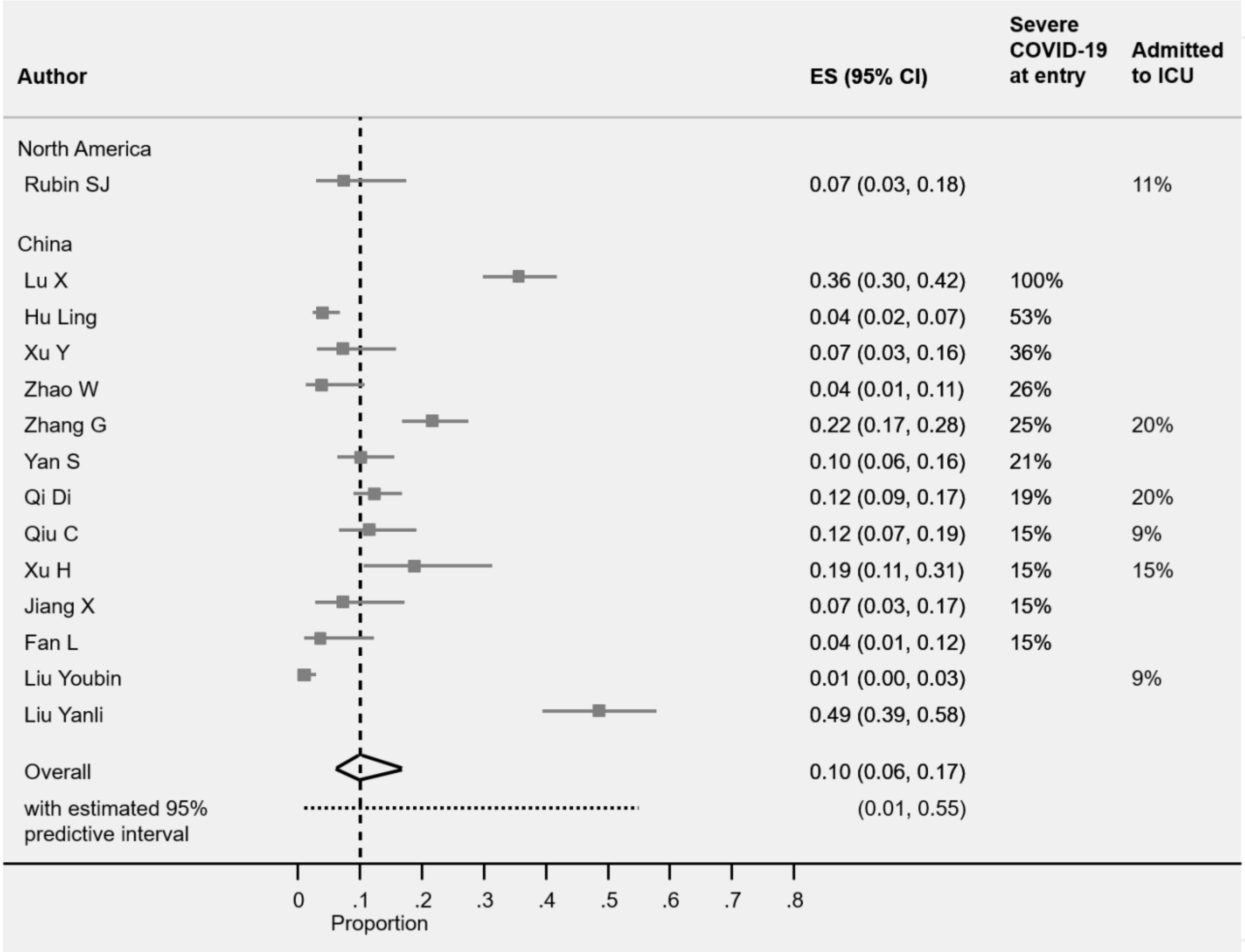

ARDS, acute respiratory distress syndrome; CI, confidence intervals; COVID-19, *Coronavirus* disease 2019; ES, estimates; ICU, intensive care unite

Figure S7. Acute cardiac injury in patients with COVID-19, not peer-reviewed studies

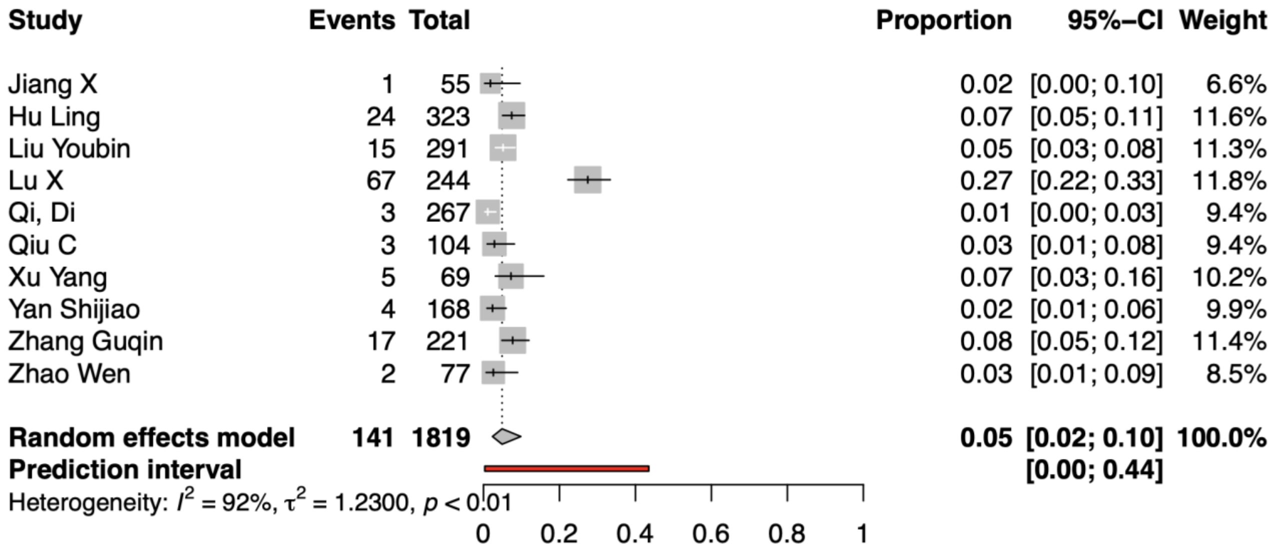

CI, confidence intervals; COVID-19, *Coronavirus* disease 2019

Figure S8. Acute kidney injury in patients with COVID-19, not peer-reviewed studies

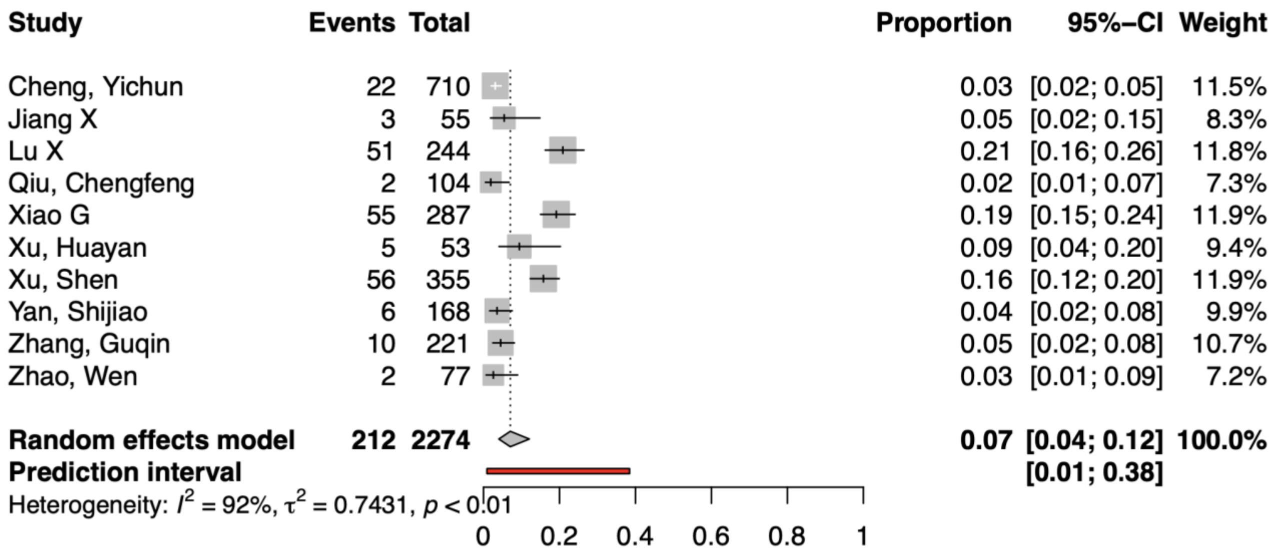

CI, confidence intervals; COVID-19, *Coronavirus* disease 2019

Figure S9. Coagulopathy in patients with COVID-19, not peer-reviewed studies

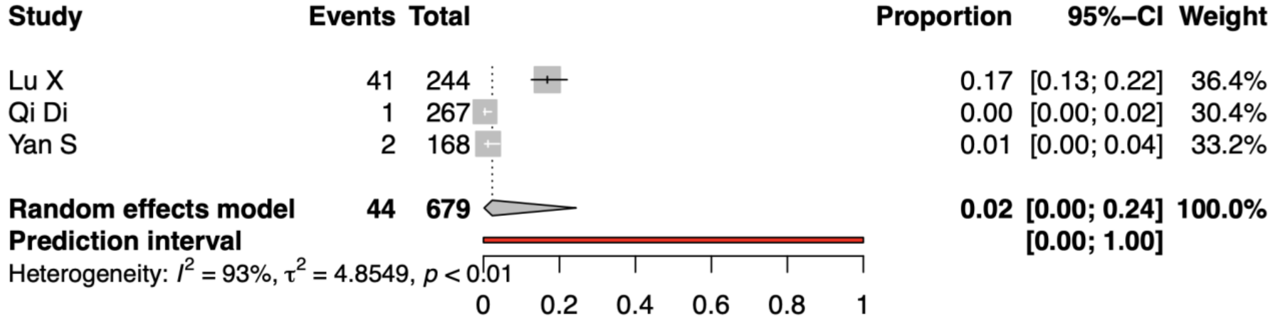

CI, confidence intervals; COVID-19, *Coronavirus* disease 2019

Figure S10. Shock in patients with COVID-19, not peer-reviewed studies

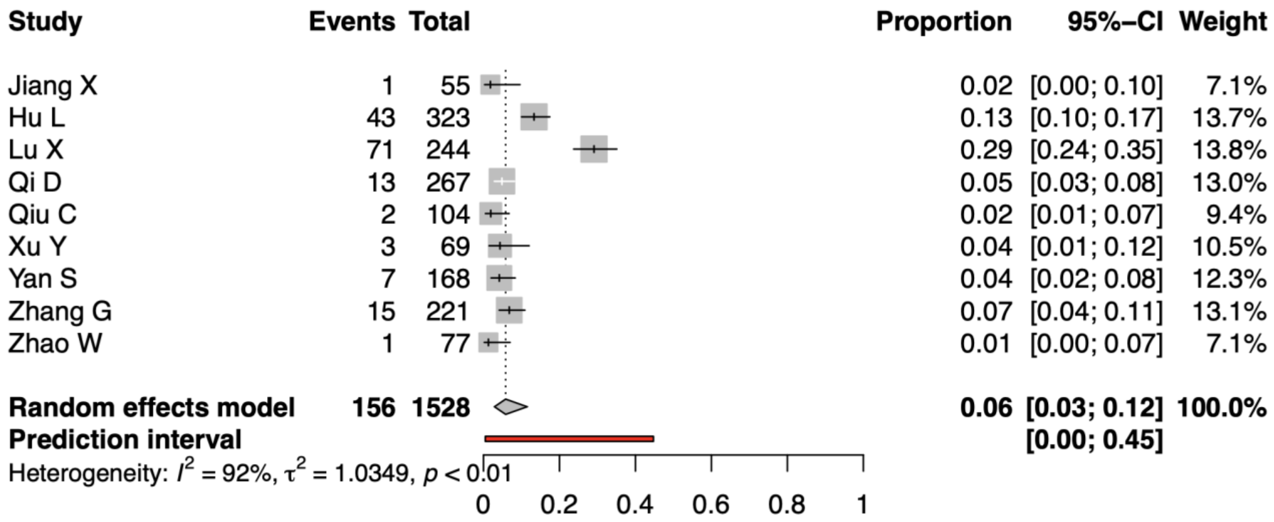

CI, confidence intervals; COVID-19, *Coronavirus* disease 2019

References of peer-reviewed studies

1. Cao B, Wang Y, Wen D, Liu W, Wang J, Fan G, et al. A Trial of Lopinavir-Ritonavir in Adults Hospitalized with Severe Covid-19. N Engl J Med. 2020.

2. Cao J, Tu WJ, Cheng W, Yu L, Liu YK, Hu X, et al. Clinical Features and Short-term Outcomes of 102 Patients with Corona Virus Disease 2019 in Wuhan, China. Clin Infect Dis. 2020.

3. Chen J, Qi T, Liu L, Ling Y, Qian Z, Li T, et al. Clinical progression of patients with COVID-19 in Shanghai, China. J Infect. 2020.

4. Chen N, Zhou M, Dong X, Qu J, Gong F, Han Y, et al. Epidemiological and clinical characteristics of 99 cases of 2019 novel coronavirus pneumonia in Wuhan, China: a descriptive study. Lancet. 2020;395(10223):507-13.

5. Chen T, Dai Z, Mo P, Li X, Ma Z, Song S, et al. Clinical characteristics and outcomes of older patients with coronavirus disease 2019 (COVID-19) in Wuhan, China (2019): a single-centered, retrospective study. J Gerontol A Biol Sci Med Sci. 2020.

6. Cui S, Chen S, Li X, Liu S, Wang F. Prevalence of venous thromboembolism in patients with severe novel coronavirus pneumonia. J Thromb Haemost. 2020.

7. Fan Z, Chen L, Li J, Cheng X, Jingmao Y, Tian C, et al. Clinical Features of COVID-19-Related Liver Damage. Clin Gastroenterol Hepatol. 2020.

8. Feng Y, Ling Y, Bai T, Xie Y, Huang J, Li J, et al. COVID-19 with Different Severity: A Multi-center Study of Clinical Features. Am J Respir Crit Care Med. 2020.

9. Gao L, Jiang D, Wen XS, Cheng XC, Sun M, He B, et al. Prognostic value of NT-proBNP in patients with severe COVID-19. Respir Res. 2020;21(1):83.

10. Grasselli G, Zangrillo A, Zanella A, Antonelli M, Cabrini L, Castelli A, et al. Baseline Characteristics and Outcomes of 1591 Patients Infected With SARS-CoV-2 Admitted to ICUs of the Lombardy Region, Italy. JAMA. 2020.

11. Grein J, Ohmagari N, Shin D, Diaz G, Asperges E, Castagna A, et al. Compassionate Use of Remdesivir for Patients with Severe Covid-19. N Engl J Med. 2020.

12. Guan WJ, Liang WH, Zhao Y, Liang HR, Chen ZS, Li YM, et al. Comorbidity and its impact on 1590 patients with Covid-19 in China: A Nationwide Analysis. Eur Respir J. 2020.

13. Guan WJ, Ni ZY, Hu Y, Liang WH, Ou CQ, He JX, et al. Clinical Characteristics of Coronavirus Disease 2019 in China. N Engl J Med. 2020.

14. Guo T, Fan Y, Chen M, Wu X, Zhang L, He T, et al. Cardiovascular Implications of Fatal Outcomes of Patients With Coronavirus Disease 2019 (COVID-19). JAMA Cardiol. 2020.

15. Han H, Xie L, Liu R, Yang J, Liu F, Wu K, et al. Analysis of heart injury laboratory parameters in 273 COVID-19 patients in one hospital in Wuhan, China. J Med Virol. 2020.

16. Jin X, Lian JS, Hu JH, Gao J, Zheng L, Zhang YM, et al. Epidemiological, clinical and virological characteristics of 74 cases of coronavirus-infected disease 2019 (COVID-19) with gastrointestinal symptoms. Gut. 2020.

17. Klok FA, Kruip M, van der Meer NJM, Arbous MS, Gommers D, Kant KM, et al. Incidence of thrombotic complications in critically ill ICU patients with COVID-19. Thromb Res. 2020.

18. Li R, Tian J, Yang F, Lv L, Yu J, Sun G, et al. Clinical characteristics of 225 patients with COVID-19 in a tertiary Hospital near Wuhan, China. J Clin Virol. 2020;127:104363.

19. Li X, Xu S, Yu M, Wang K, Tao Y, Zhou Y, et al. Risk factors for severity and mortality in adult COVID-19 inpatients in Wuhan. J Allergy Clin Immunol. 2020.

20. Lian J, Jin X, Hao S, Cai H, Zhang S, Zheng L, et al. Analysis of Epidemiological and Clinical features in older patients with Corona Virus Disease 2019 (COVID-19) out of Wuhan. Clin Infect Dis. 2020.

21. Liu K, Chen Y, Lin R, Han K. Clinical features of COVID-19 in elderly patients: A comparison with young and middle-aged patients. J Infect. 2020.

22. Liu K, Fang YY, Deng Y, Liu W, Wang MF, Ma JP, et al. Clinical characteristics of novel coronavirus cases in tertiary hospitals in Hubei Province. Chin Med J (Engl). 2020.

23. Liu W, Tao ZW, Lei W, Ming-Li Y, Kui L, Ling Z, et al. Analysis of factors associated with disease outcomes in hospitalized patients with 2019 novel coronavirus disease. Chin Med J (Engl). 2020.

24. Qian GQ, Yang NB, Ding F, Ma AHY, Wang ZY, Shen YF, et al. Epidemiologic and Clinical Characteristics of 91 Hospitalized Patients with COVID-19 in Zhejiang, China: A retrospective, multi-centre case series. QJM. 2020.

25. Shi H, Han X, Jiang N, Cao Y, Alwalid O, Gu J, et al. Radiological findings from 81 patients with COVID-19 pneumonia in Wuhan, China: a descriptive study. Lancet Infect Dis. 2020;20(4):425-34.

26. Shi S, Qin M, Shen B, Cai Y, Liu T, Yang F, et al. Association of Cardiac Injury With Mortality in Hospitalized Patients With COVID-19 in Wuhan, China. JAMA Cardiol. 2020.

27. Tang N, Bai H, Chen X, Gong J, Li D, Sun Z. Anticoagulant treatment is associated with decreased mortality in severe coronavirus disease 2019 patients with coagulopathy. J Thromb Haemost. 2020;18(5):1094-9.

28. Tang N, Li D, Wang X, Sun Z. Abnormal coagulation parameters are associated with poor prognosis in patients with novel coronavirus pneumonia. J Thromb Haemost. 2020;18(4):844-7.

29. Tian S, Hu N, Lou J, Chen K, Kang X, Xiang Z, et al. Characteristics of COVID-19 infection in Beijing. J Infect. 2020;80(4):401-6.

30. Wang D, Hu B, Hu C, Zhu F, Liu X, Zhang J, et al. Clinical Characteristics of 138 Hospitalized Patients With 2019 Novel Coronavirus-Infected Pneumonia in Wuhan, China. JAMA. 2020.

31. Wang L, He W, Yu X, Hu D, Bao M, Liu H, et al. Coronavirus disease 2019 in elderly patients: Characteristics and prognostic factors based on 4-week follow-up. J Infect. 2020.

32. Wang L, Li X, Chen H, Yan S, Li D, Li Y, et al. Coronavirus Disease 19 Infection Does Not Result in Acute Kidney Injury: An Analysis of 116 Hospitalized Patients from Wuhan, China. Am J Nephrol. 2020:1-6.

33. Wang Y, Lu X, Chen H, Chen T, Su N, Huang F, et al. Clinical Course and Outcomes of 344 Intensive Care Patients with COVID-19. Am J Respir Crit Care Med. 2020.

34. Wang Y, Dong C, Hu Y, Li C, Ren Q, Zhang X, et al. Temporal Changes of CT Findings in 90 Patients with COVID-19 Pneumonia: A Longitudinal Study. Radiology. 2020:200843.

35. Wang Z, Yang B, Li Q, Wen L, Zhang R. Clinical Features of 69 Cases with Coronavirus Disease 2019 in Wuhan, China. Clin Infect Dis. 2020.

36. Wu C, Chen X, Cai Y, Xia J, Zhou X, Xu S, et al. Risk Factors Associated With Acute Respiratory Distress Syndrome and Death in Patients With Coronavirus Disease 2019 Pneumonia in Wuhan, China. JAMA Intern Med. 2020.

37. Wu J, Liu J, Zhao X, Liu C, Wang W, Wang D, et al. Clinical Characteristics of Imported Cases of COVID-19 in Jiangsu Province: A Multicenter Descriptive Study. Clin Infect Dis. 2020.

38. Xu XW, Wu XX, Jiang XG, Xu KJ, Ying LJ, Ma CL, et al. Clinical findings in a group of patients infected with the 2019 novel coronavirus (SARS-Cov-2) outside of Wuhan, China: retrospective case series. BMJ. 2020;368:m606.

39. Yang W, Cao Q, Qin L, Wang X, Cheng Z, Pan A, et al. Clinical characteristics and imaging manifestations of the 2019 novel coronavirus disease (COVID-19):A multi-center study in Wenzhou city, Zhejiang, China. J Infect. 2020;80(4):388-93.

40. Yang X, Yu Y, Xu J, Shu H, Xia J, Liu H, et al. Clinical course and outcomes of critically ill patients with SARS-CoV-2 pneumonia in Wuhan, China: a single-centered, retrospective, observational study. Lancet Respir Med. 2020.

41. Yang X, Yang Q, Wang Y, Wu Y, Xu J, Yu Y, et al. Thrombocytopenia and Its Association with Mortality in Patients with COVID-19. J Thromb Haemost. 2020.

42. Zhang J, Wang X, Jia X, Li J, Hu K, Chen G, et al. Risk factors for disease severity, unimprovement, and mortality in COVID-19 patients in Wuhan, China. Clin Microbiol Infect. 2020.

43. Zhang L, Yan X, Fan Q, Liu H, Liu X, Liu Z, et al. D-dimer levels on admission to predict in-hospital mortality in patients with Covid-19. J Thromb Haemost. 2020.

44. Zhou F, Yu T, Du R, Fan G, Liu Y, Liu Z, et al. Clinical course and risk factors for mortality of adult inpatients with COVID-19 in Wuhan, China: a retrospective cohort study. Lancet. 2020;395(10229):1054-62.

References of not peer-reviewed studies

45. Benelli G, Buscarini E, Canetta C, La Piana G, Merli GJ, Scartabellati A, et al. SARS-COV2 comorbidity network and outcome in hospitalized patients in Crema, Italy. doi: 10.1101/2020.04.14.20053090.

46. Chen C, Huang J, Cheng Z, Wu J, Chen S, Zhang Y, et al. Favipiravir versus Arbidol for COVID-19: A Randomized Clinical Trial. doi: 10.1101/2020.03.17.20037432.

47. Chen X, Jiang Q, Ma Z, Ling J, Hu W, Cao Q, et al. Clinical Characteristics Hospitalized Patients with SARS-Cov-2 and HBV Co- infection. doi: 10.1101/2020.03.23.20040733.

48. Chen X, Zheng F, Qing Y, Ding S, Yang D, Lei C, et al. Epidemiological and clinical features of 291 cases with coronavirus disease 2019 in areas adjacent to Hubei, China: a double-center observational study. doi: 10.1101/2020.03.03.20030353.

49. Cheng Y, Luo R, Wang K, Zhang W, Wang Z, Dong L, et al. Kidney impairment is associated with in-hospital death of COVID-19 patients. doi: 10.1101/2020.02.18.20023242.

50. Cummings M, Baldwin M, Abrams D, Jacobson S, Meyer B, Balough E, et al. Epidemiology, clinical course, and outcomes of critically ill adults with COVID-19 in New York City: a prospective cohort study. doi: 10.1101/2020.04.15.20067157.

51. Fan L, Liu H, Li N, Liu C, Gu Y, Liu Y, et al. Medical treatment of 55 patients with COVID-19 from seven cities in northeast China who fully recovered: a single-center, retrospective, observational study. doi: 10.1101/2020.03.28.20045955.

52. Feng Z, Li J, Yao S, Yu Q, Zhou W, Mao X, et al. The Use of Adjuvant Therapy in Preventing Progression to Severe Pneumonia in Patients with Coronavirus Disease 2019: A Multicenter Data Analysis. doi: 10.1101/2020.04.08.20057539.

53. Fu L, Fei J, Xu S, Xiang H, Xiang Y, Tan Z, et al. Acute liver injury and its association with death risk of patients with COVID-19: a hospital-based prospective case-cohort study. doi: 10.1101/2020.04.02.20050997.

54. Giorgi Rossi P, Marino M, Formisano D, Venturelli F, Vicentini M, Grilli R, et al. Title: Characteristics and outcomes of a cohort of SARS-CoV-2 patients in the Province of Reggio Emilia, Italy. doi: 10.1101/2020.04.13.20063545.

55. Hu L, Chen S, Fu Y, Gao Z, Long H, Ren H, et al. Risk Factors Associated with Clinical Outcomes in 323 COVID-19 Patients in Wuhan, China. doi: 10.1101/2020.03.25.20037721.

56. Jiang X, Tao J, Wu H, Wang Y, Zhao W, Zhou M, et al. Clinical features and management of severe COVID-19: A retrospective study in Wuxi, Jiangsu Province, China. doi: 10.1101/2020.04.10.20060335.

57. Joharatnam-Hogan N, Hochhauser D, Shiu K, Rush H, Crolley V, Butcher E, et al. Outcomes of the 2019 Novel Coronavirus in patients with or without a history of cancer - a multi-centre North London experience. doi: 10.1101/2020.04.16.20061127.

58. Liu J, Ouyang L, Guo P, Wu H, Fu P, Chen Y, et al. Epidemiological, Clinical Characteristics and Outcome of Medical Staff Infected with COVID-19 in Wuhan, China: A Retrospective Case Series Analysis. doi: 10.1101/2020.03.09.20033118.

59. Liu Q, Fang X, Tian L, Chung U, Chen X, Wang K, et al. The effect of Arbidol Hydrochloride on reducing mortality of Covid-19 patients: a retrospective study of real-world data from three hospitals in Wuhan. doi: 10.1101/2020.04.11.20056523.

60. Liu Q, Fang X, Tokuno S, Chung U, Chen X, Dai X, et al. Prediction of the clinical outcome of COVID-19 patients using T lymphocyte subsets with 340 cases from Wuhan, China: a retrospective cohort study and a web visualization tool. doi: 10.1101/2020.04.06.20056127.

61. Liu Y, Sun W, Li J, Chen L, Wang Y, Zhang L, et al. Clinical features and progression of acute respiratory distress syndrome in coronavirus disease 2019. doi: 10.1101/2020.02.17.20024166.

62. Liu Y, Li J, Liu D, Song H, Chen C, Iv M, et al. Clinical features and outcomes of 2019 novel coronavirus-infected patients with cardiac injury. doi: 10.1101/2020.03.11.20030957.

63. Lu X, Chen T, Wang Y, Wang J, Zhang B, Li Y, et al. Adjuvant corticosteroid therapy for critically ill patients with COVID-19. doi: 10.1101/2020.04.07.20056390.

64. Magagnoli J, Narendran S, Pereira F, Cummings T, Hardin J, Sutton S, et al. Outcomes of hydroxychloroquine usage in United States veterans hospitalized with Covid-19. doi: 10.1101/2020.04.16.20065920.

65. Qi D, Yan X, Tang X, Peng J, Yu Q, Feng L, et al. Epidemiological and clinical features of 2019-nCoV acute respiratory disease cases in Chongqing municipality, China: a retrospective, descriptive, multiple-center study. doi: 10.1101/2020.03.01.20029397.

66. Qiu C, Xiao Q, Liao X, Deng Z, Liu H, Shu Y, et al. Transmission and clinical characteristics of coronavirus disease 2019 in 104 outside-Wuhan patients, China. doi: 10.1101/2020.03.04.20026005.

67. Rubin J, Falkson S, Degner N, Blish C. Clinical characteristics associated with COVID-19 severity in California. doi: 10.1101/2020.03.27.20043661.

68. Wang Z, Ji J, Liu Y, Liu R, Zha Y, Chang X, et al. Survival analysis of hospital length of stay of novel coronavirus (COVID-19) pneumonia patients in Sichuan, China. doi: 10.1101/2020.04.07.20057299.

69. Wen Y, Wei L, Li Y, Tang X, Feng S, Leung K, et al. Epidemiological and clinical characteristics of COVID-19 in Shenzhen, the largest migrant city of China. doi: 10.1101/2020.03.22.20035246.

70. Wu C, Hu X, Song J, Du C, Xu J, Yang D, et al. Heart injury signs are associated with higher and earlier mortality in coronavirus disease 2019 (COVID-19). doi: 10.1101/2020.02.26.20028589.

71. Wu Y, Guo W, Liu H, Qi B, Liang K, Xu B, et al. Clinical outcomes of 402 patients with COVID-2019 from a single center in Wuhan, China. doi: 10.1101/2020.03.07.20032672.

72. Xiao G, Hu H, Wu F, Sha T, Huang Q, Li H, et al. Acute kidney injury in patients hospitalized with COVID-19 in Wuhan, China: A single-center retrospective observational study. doi: 10.1101/2020.04.06.20055194.

73. Xu H, Hou K, Xu H, Li Z, Chen H, Zhang N, et al. Acute Myocardial Injury of Patients with Coronavirus Disease 2019. doi: 10.1101/2020.03.05.20031591.

74. Xu S, Fu L, Fei J, Xiang H, Xiang Y, Tan Z, et al. Acute kidney injury at early stage as a negative prognostic indicator of patients with COVID-19: a hospital-based retrospective analysis. doi: 10.1101/2020.03.24.20042408.

75. Xu Y, Li Y, Zeng Q, Lu Z, Li Y, Wu W, et al. Clinical Characteristics of SARS-CoV-2 Pneumonia Compared to Controls in Chinese Han Population. doi: 10.1101/2020.03.08.20031658.

76. Yan S, Song X, Lin F, Zhu H, Wang X, Li M, et al. Clinical Characteristics of Coronavirus Disease 2019 in Hainan, China. doi: 10.1101/2020.03.19.20038539.

77. Zeng L, Li J, Liao M, Hua R, Huang P, Zhang M, et al. Risk assessment of progression to severe conditions for patients with COVID-19 pneumonia: a single-center retrospective study. doi: 10.1101/2020.03.25.20043166.

78. Zhang G, Hu C, Luo H, Fang F, Chen Y, Li J, et al. Clinical features and outcomes of 221 patients with COVID-19 in Wuhan, China. doi: 10.1101/2020.03.02.20030452.

79. Zhao W, Yu S, Zha X, Wang N, Pang Q, Li T, et al. Clinical characteristics and durations of hospitalized patients with COVID-19 in Beijing: a retrospective cohort study. doi: 10.1101/2020.03.13.20035436.

PRISMA 2009 checklist

Section/topic	#	Checklist item	Reported on page #
**TITLE**			
Title	1	Identify the report as a systematic review, meta-analysis, or both.	1
**ABSTRACT**			
Structured summary	2	Provide a structured summary including, as applicable: background; objectives; data sources; study eligibility criteria, participants, and interventions; study appraisal and synthesis methods; results; limitations; conclusions and implications of key findings; systematic review registration number.	2-3
**INTRODUCTION**			
Rationale	3	Describe the rationale for the review in the context of what is already known.	4
Objectives	4	Provide an explicit statement of questions being addressed with reference to participants, interventions, comparisons, outcomes, and study design (PICOS).	4
**METHODS**			
Protocol and registration	5	Indicate if a review protocol exists, if and where it can be accessed (e.g., Web address), and, if available, provide registration information including registration number.	4
Eligibility criteria	6	Specify study characteristics (e.g., PICOS, length of follow-up) and report characteristics (e.g., years considered, language, publication status) used as criteria for eligibility, giving rationale.	5
Information sources	7	Describe all information sources (e.g., databases with dates of coverage, contact with study authors to identify additional studies) in the search and date last searched.	4-5, Additional file 1
Search	8	Present full electronic search strategy for at least one database, including any limits used, such that it could be repeated.	Additional file 1
Study selection	9	State the process for selecting studies (i.e., screening, eligibility, included in systematic review, and, if applicable, included in the meta-analysis).	5
Data collection process	10	Describe method of data extraction from reports (e.g., piloted forms, independently, in duplicate) and any processes for obtaining and confirming data from investigators.	5
Data items	11	List and define all variables for which data were sought (e.g., PICOS, funding sources) and any assumptions and simplifications made.	5-6
Risk of bias in individual studies	12	Describe methods used for assessing risk of bias of individual studies (including specification of whether this was done at the study or outcome level), and how this information is to be used in any data synthesis.	6
Summary measures	13	State the principal summary measures (e.g., risk ratio, difference in means).	7
Synthesis of results	14	Describe the methods of handling data and combining results of studies, if done, including measures of consistency (e.g., I^2) for each meta-analysis.	7-8

Section/topic	#	Checklist item	Reported on page #
Risk of bias across studies	15	Specify any assessment of risk of bias that may affect the cumulative evidence (e.g., publication bias, selective reporting within studies).	-
Additional analyses	16	Describe methods of additional analyses (e.g., sensitivity or subgroup analyses, meta-regression), if done, indicating which were pre-specified.	7-8
**RESULTS**			
Study selection	17	Give numbers of studies screened, assessed for eligibility, and included in the review, with reasons for exclusions at each stage, ideally with a flow diagram.	8
Study characteristics	18	For each study, present characteristics for which data were extracted (e.g., study size, PICOS, follow-up period) and provide the citations.	8-9, Additional file 1
Risk of bias within studies	19	Present data on risk of bias of each study and, if available, any outcome level assessment (see item 12).	9, Additional file 1
Results of individual studies	20	For all outcomes considered (benefits or harms), present, for each study: (a) simple summary data for each intervention group (b) effect estimates and confidence intervals, ideally with a forest plot.	10-11
Synthesis of results	21	Present results of each meta-analysis done, including confidence intervals and measures of consistency.	10-11
Risk of bias across studies	22	Present results of any assessment of risk of bias across studies (see Item 15).	-
Additional analysis	23	Give results of additional analyses, if done (e.g., sensitivity or subgroup analyses, meta-regression [see Item 16]).	11, Additional file 1
**DISCUSSION**			
Summary of evidence	24	Summarize the main findings including the strength of evidence for each main outcome; consider their relevance to key groups (e.g., healthcare providers, users, and policy makers).	12-13
Limitations	25	Discuss limitations at study and outcome level (e.g., risk of bias), and at review-level (e.g., incomplete retrieval of identified research, reporting bias).	13
Conclusions	26	Provide a general interpretation of the results in the context of other evidence, and implications for future research.	14
**FUNDING**			
Funding	27	Describe sources of funding for the systematic review and other support (e.g., supply of data); role of funders for the systematic review.	15
